# Immunoglobulins G from patients with ANCA-associated vasculitis are atypically glycosylated in both the Fc and Fab regions and the relation to disease activity

**DOI:** 10.1371/journal.pone.0213215

**Published:** 2019-02-28

**Authors:** Olivier M. Lardinois, Leesa J. Deterding, Jacob J. Hess, Caroline J. Poulton, Candace D. Henderson, J. Charles Jennette, Patrick H. Nachman, Ronald J. Falk

**Affiliations:** 1 UNC Kidney Center, Division of Nephrology and Hypertension, Department of Medicine, University of North Carolina at Chapel Hill, Chapel Hill, North Carolina, United States of America; 2 Mass Spectrometry Research and Support Group, National Institute of Environmental Health Sciences, NIH, Research Triangle Park, North Carolina, United States of America; 3 Department of Pathology and Laboratory of Medicine, University of North Carolina at Chapel Hill, Chapel Hill, North Carolina, United States of America; 4 Division of Renal Diseases and Hypertension, Department of Medicine, University of Minnesota, Minneapolis, Minnesota, United States of America; National Cancer Institute at Frederick, UNITED STATES

## Abstract

**Background:**

Anti-neutrophil cytoplasmic autoantibodies (ANCA) directed against myeloperoxidase (MPO) and proteinase 3 (PR3) are pathogenic in ANCA-associated vasculitis (AAV). The respective role of IgG Fc and Fab glycosylation in mediating ANCA pathogenicity is incompletely understood. Herein we investigate in detail the changes in Fc and Fab glycosylation in MPO-ANCA and Pr3-ANCA and examine the association of glycosylation aberrancies with disease activity.

**Methodology:**

Total IgG was isolated from serum or plasma of a cohort of 30 patients with AAV (14 MPO-ANCA; 16 PR3-ANCA), and 19 healthy control subjects. Anti-MPO specific IgG was affinity-purified from plasma of an additional cohort of 18 MPO-ANCA patients undergoing plasmapheresis. We used lectin binding assays, liquid chromatography, and mass spectrometry-based methods to analyze Fc and Fab glycosylation, the degree of sialylation of Fc and Fab fragments and to determine the exact localization of *N*-glycans on Fc and Fab fragments.

**Principal findings:**

IgG_1_ Fc glycosylation of total IgG was significantly reduced in patients with active AAV compared to controls. Clinical remission was associated with complete glycan normalization for PR3-ANCA patients but not for MPO-ANCA patients. Fc-glycosylation of anti-MPO specific IgG was similar to total IgG purified from plasma. A major fraction of anti-MPO specific IgG harbor extensive glycosylation within the variable domain on the Fab portion.

**Conclusions/Significance:**

Significant differences exist between MPO and PR3-ANCA regarding the changes in amounts and types of glycans on Fc fragment and the association with disease activity. These differences may contribute to significant clinical difference in the disease course observed between the two diseases.

## Introduction

Anti-neutrophil cytoplasmic autoantibody (ANCA)-vasculitis (AAV) is a systemic autoimmune disease characterized by focal necrotizing lesions affecting arterioles, capillaries, and venules. This disease may affect many organs, and the kidneys and the lungs are commonly involved [1, 2]. The two major ANCA antigens are myeloperoxidase (MPO) [3] and proteinase 3 (PR3) [4], which are constituents of neutrophil primary granules [5] and monocyte lysosomes [6]. PR3-ANCA are found in the majority of patients with granulomatosis with polyangiitis (GPA), and some patients with and microscopic polyangiitis (MPA). MPO-ANCA are found in most patients with MPA as well as in some patients with GPA or with eosinophilic GPA (EGPA). There is increasing support to categorize patients based on their ANCA serotype, *i*.*e*., PR3-ANCA and MPO-ANCA, as opposed to the traditional disease classification, *i*.*e*., GPA, MPA, and EGPA, as significant clinical differences exist between these two small-vessel vasculitides [7–17].

ANCA are pathogenic [18–20] as a consequence of ANCA binding to their target antigens and engagement of Fc-gamma receptor (FcγR) on the surface of neutrophils and monocytes. This process leads to endothelial adhesion, production of reactive oxygen and nitrogen species, degranulation and associated release of proteolytic enzymes, which are injurious to endothelial cells [21].

**Fig 1:**
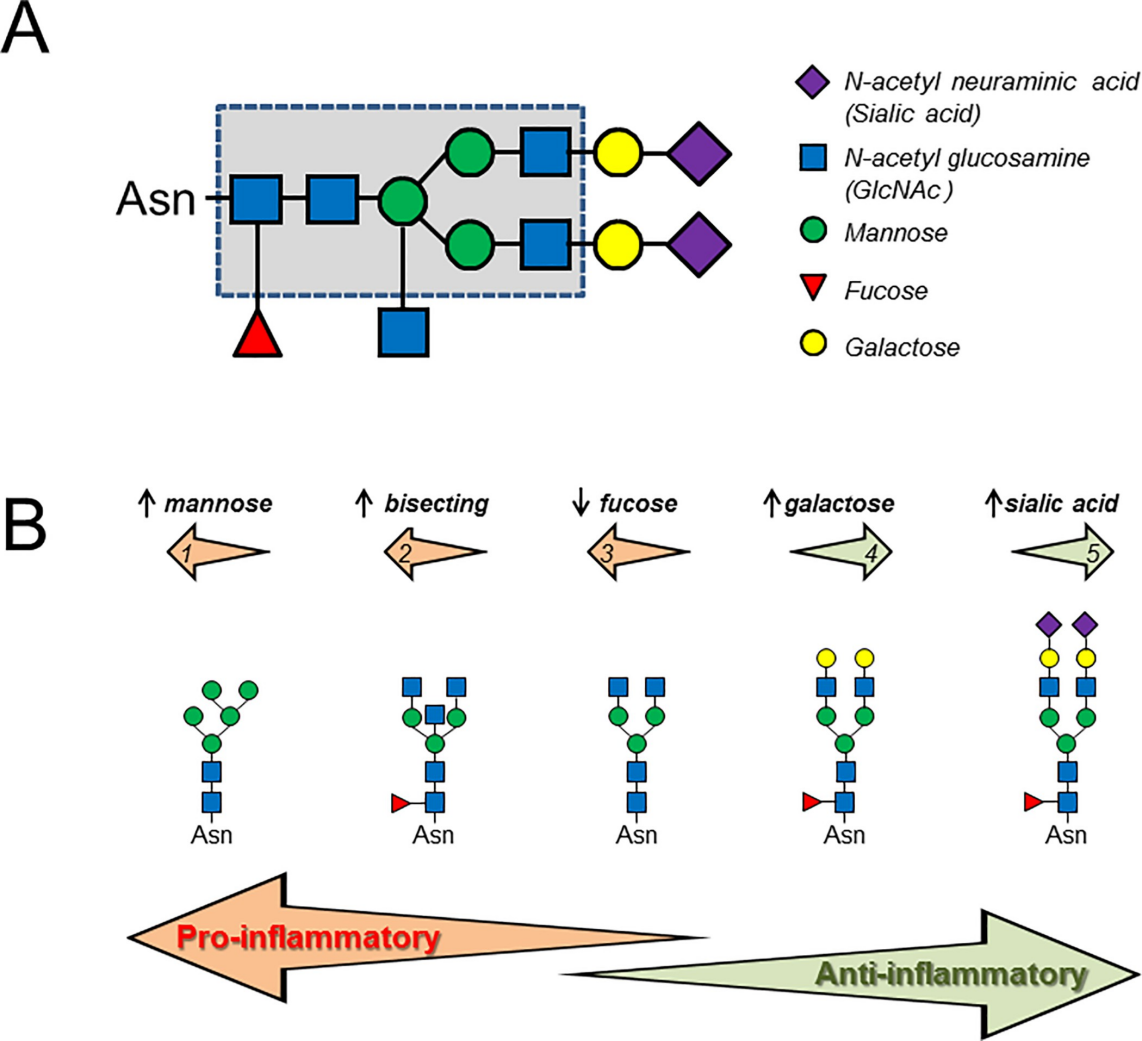
Schematic picture showing IgG Fc *N*-linked glycans heterogeneity and their downstream effects. A: The *N*-glycans attached at position Asn297 of IgG Fc fragment are predominantly core-fucosylated bi-antennary structures of the complex type with the arms terminating either with *N*-acetylglucosamine (Glc*N*Ac) or *N*-acetylglucosamine-galactose (Glc*N*Ac-Gal). The dashed line indicates the conserved heptasaccharide core with possible extensions. B: IgG Fc glycosylation, simplified as five representative structures, can significantly impact effector functions of the IgG molecules. High-mannose *N*-glycans, bisecting *N*-acetyl glucosamine (GlcNAc), or absence of core fucose, act mainly pro-inflammatory (arrows 1, 2, and 3, respectively); terminal galactose or sialic residues (*N*-acetylneuraminic acid) act mainly anti-inflammatory (arrows 4 and 5, respectively). Symbols and colors are drawn according to the Consortium for Functional Glycomics.

ANCA are usually of IgG isotype, and predominantly belong to the IgG1 and IgG4 subclasses [22]. The four human IgG subclasses contain an *N*-glycan attached to the highly conserved Asn297 residue of the Fc region. The *N*-glycans attached to this site are predominantly bi-antennary structures of the complex type that vary with respect to the presence of a core fucose and to the degree of sialylation (*N*-acetyl neuraminic acid) and galactosylation (Fig 1A). Small amounts of these *N*-glycans comprise tri-antennary structures of the complex type with bisecting *N*-acetyl glucosamine (GlcNAc) or structures of the high-mannose type. The *N*-glycans attached at position Asn297 determine the ability of the IgG to interact with various cellular receptors expressed by innate and adaptive immune cells or the complement-activating protein mannose-binding lectin (MBL) [23–32] (Fig 1B). For example, glycoforms lacking terminal galactose residues have an enhanced pro-inflammatory activity, whereas the addition of galactose decreases their inflammatory potential [23–25]. The addition of sialic acid changes the physiological role of IgG from pro-inflammatory to anti-inflammatory agents by modulating interaction with the lectins DC-SIGN and Dectin-1 [25, 28]. Core fucose at the Fc region interferes with the binding of IgG to FcγRIIIa and diminishes antibody-dependent cell-mediated cytotoxicity (ADCC) [29, 30], whereas deletion of the core fucose results in enhanced cytotoxicity [33]. Conversely, complete removal of the Fc glycans by glycoside hydrolases abolishes ADCC and complement-dependent cytotoxicity (CDC) [32].

The variable domains of IgG may also contain *N*-linked glycans. Estimates of the percentage of Fab-glycosylated IgG in healthy individuals range from ~15 to 25% [34]. Compared with IgG Fc glycans, IgG Fab glycans contain high percentages of bisecting GlcNAc, galactose, sialic acid and low percentages of core fucose [35–37]. Limited information is available regarding the role of *N*-glycosylation in the variable domain. This is, in part, due to technical challenges in detecting the presence of *N*-linked glycans on the IgG Fab portion [35]. Fab *N*-glycans increase the affinity and avidity for antigens of some, but not all antibodies [38–40], whereas glycosylation may affect the serum half-life of some antibodies [41].

Although glycosylation status of ANCA is thought to be important for their pathogenic potential, the respective roles of IgG Fc and Fab glycosylation in ANCA pathogenicity are still largely unexplored. In the current study, we used lectin binding assays, liquid chromatography, and mass spectrometry-based methods to analyze Fc and Fab glycosylation, the degree of sialylation of Fc and Fab fragments and to determine the exact localization of *N*-glycans on Fc and Fab fragments. These analyses show significant differences between MPO- and PR3-ANCA diseases regarding the changes in amounts and types of glycans on Fc portions of total IgG that is associated with disease activity. We also demonstrate that anti-MPO IgG from patients with active ANCA are both hypoglycosylated in the Fc region and hyperglycosylated in the Fab region. We provide evidence that the additional glycans in the Fab region of MPO-ANCA have been introduced most likely by somatic hypermutation.

## Materials and methods

Detailed information about polyclonal IgG purification, generation of IgG-F(ab’)_2_ and IgG-Fc fragments, pull-down experiments using SNA and MPO-coated beads, enzyme-linked lectin assay (ELLA), anti-MPO enzyme-linked immunosorbent assay (ELISA), and Lectin blot analysis are given in the supplemental material and methods (See S1 Text in the Supporting Information).

### Patient and control samples

Serum or plasma from freshly drawn blood were obtained from patients with ANCA-associated vasculitis (AAV) with biopsy-proven necrotizing and crescentic glomerulonephritis or from control subjects with no history of kidney disease. Plasma from the first plasmapheresis treatment of patients with AAV was used for affinity isolation of anti-MPO autoantibodies. Informed consent was obtained in accordance with our institutional review board’s guidelines for human participants. All patients fulfilled the Chapel Hill Consensus Conference nomenclature definition of ANCA vasculitis [42]. Vasculitis disease activity was measured using the Birmingham Vasculitis Activity Score (BVAS) [43]. Blood samples from each patient were collected at both active and remission stages. Every patient with active vasculitis had a BVAS ≥ 3 at screening. When possible, “active” samples were obtained at disease onset; otherwise, the sample corresponding to the highest BVAS score was used in these analyses. Samples were classified as “remission” if patients had a BVAS = 0 for 3 months before and after the collection date.

### Analysis of glycopeptides

#### LC-MS analysis of glycopeptides

LC-MS was performed on a Waters-Micromass Q-Tof Premier mass spectrometer equipped with a nanoAcquity UPLC system (Waters). Analyses were performed on a 1.8 μm, 75 μm × 150 mm, HSS T3 column (Waters, nanoAcquity), using a flow rate of 300 nL/min. A C18 trapping column (180 μm × 20 mm) with 5 μm particle size (Waters, nanoAcquity) was positioned in line with the analytical column and upstream of a micro-tee union used both as a vent for trapping and as a liquid junction. Trapping was performed for 3 min at a 5 μL/min flow rate, using the initial solvent composition. A 10 μL aliquot of the digest sample was injected onto the column. Peptides were eluted by using a linear gradient from 98% solvent A (0.1% formic acid in water (v/v)) and 2% solvent B (0.1% formic acid in acetonitrile (v/v)) to 40% solvent B over 90 min. The mass spectra were acquired over the mass range 200–2000 Da. A capillary voltage of 3.2 kV and a cone voltage of 20 V were used for glycopeptide analysis, in order to prevent in-source decomposition. For subclass-specific glycosylation analysis, the instrument was operated in the MS only mode. For calibration, an external lock mass was used with a separate reference spray (LockSpray) using a solution of Glu-Fibrinopeptide B (300 fmol/μL) in water/acetonitrile 80:20 (v/v) and 0.1% formic acid with a mass of 785.8496 (2+).

#### Data analysis

Data analysis was performed using MassLynx 4.1 software. For subclass-specific glycosylation analysis, data were averaged, and the mean and standard deviation were calculated for each glycoform of each subclass using fourteen separates experimentally-determined ion abundances. Quantification was performed by integrating and summing the monoisotopic peak and the first three ^13^C-containing isotope peaks of the double protonated species. Subclass-specific glycosylation analysis was performed by averaging the MS scans over the chromatographic retention time in which each glycopeptides from a specific subclass eluted. IgG_2_ and IgG_3_ have identical peptide moieties (E_293_EQFNSTFR_301_) of their tryptic Fc glycopeptides and are, therefore, not distinguished by the profiling method. The IgG_4_ glycopeptides were not analyzed due to low abundance. Glycosylation features were calculated [44] using the following equations: galactosylation = (G_1_ + G_1_F + G_1_N + G_1_FN + G_1_FS) × 0.5 + G_2_ + G_2_F + G_2_N + G_2_FN + G_2_FS; agalactosylation = G_o_ + G_o_F + G_o_N + G_o_FN; sialylation = G_1_FS + G_2_FS; bisection = G_0_N + G_o_FN + G_1_N + G_1_FN + G_2_N + G_2_FN; fucosylation = G_o_F + G_1_F + G_0_FN + G_2_F + G_1_FN + G_1_FS + G_2_FN + G_2_FS. A list of calculated m/z values for the 14 most abundant tryptic glycopeptides detected in the present study is provided in the supplementary material (S1 Table).

### Analysis of released *N*-glycans

#### Glycan release and purification of released *N-*glycans

Glycans were released by digesting the IgG samples with PNGase F (New England Biolabs) in 50 mM ammonium bicarbonate buffer, pH 7.8, for 16–24 h at 37°C. The released glycans were then isolated by solid-phase extraction of proteins on HyperSep C18 columns (Thermo Scientific). The flow-through and washes containing the glycans were dried by vacuum centrifugation prior to labeling.

#### Fluorescence labeling and chromatography

Released glycans were fluorescently labeled *via* reductive amination with 2-aminobenzoic acid (2-AB) and sodium cyanoborohydride in 30% v/v acetic acid in DMSO at 65° C for 3 hours. Excess labeling reagents and reducing agent were removed from the samples using GlycoClean S solid-phase extraction cartridges according to the manufacturer’s instructions. Hydrophilic interaction chromatography (HILIC) separation of 2-AB labeled glycans was carried out using an Agilent 1100 HPLC system coupled to an Agilent HPLC fluorescence (FLD) detector. Separations were performed using Waters BEH Glycan column, 100 mm × 2.1 mm i.d., 2.5 μm amide sorbent, with the column heated to 60° C. The injection volume was 20 μl. All separations were performed using 100 mM ammonium formate, pH 4.5, as solvent A and 100% acetonitrile as solvent B. The gradient conditions were as follows: 0–5 min, 85–75% B, 0.3 ml min^-1^; 5–35 min, 75–64% B, 0.3 ml min^-1^; 35–40 min, 64–50% B, 0.3 ml min^-1^; 40–42 min, 50–50% B, 0.3–0.1 ml min^-1^; 42–43 min, 50–10% B, 0.1 ml min^-1^; 43–48 min, 10–10% B, 0.1 ml min^-1^; 48–50 min, 10–85% B, 0.1 ml min^-1^; 50–60 min, 85–85% B, 0.1–0.3 ml min^-1^. The fluorescence detector excitation and emission wavelengths were set at 260 and 430 nm, respectively.

### Mapping *N*-linked glycosylation sites using hydrazide chemistry and mass spectrometry

#### *N*-glycopeptide enrichment by hydrazide resin capture

Hydrazide resin (Bio-Rad, Hercules, CA) and a method similar to that reported previously [45, 46] was used for capturing *N*-glycopeptides. Purified IgG samples were buffer-exchanged with 10 mM sodium acetate, 150 mM NaCl, pH 5.5 using a PD-10 column, and concentrated to a final concentration of 4.7 mg/ml, before oxidation with 15 mM sodium periodate (NaIO_4_) at room temperature for 1 hour in the dark with constant shaking. The excess NaIO_4_ was removed by performing buffer exchange with coupling buffer (10 mM sodium acetate, 1 M NaCl, pH 4.5) on a PD 10 column. The hydrazide resin (2 ml of 50% slurry per 170 μl of buffered-exchanged IgG) was washed with 5 volumes of coupling buffer. The oxidized IgG sample was then added and incubated with the resin overnight at 4° C. Nonglycoproteins were removed by washing the resin briefly three times with 6 M guanidine in 100 mM NaHCO_3_, pH 8.3.

#### On-beads digestion

The IgG samples bound to the hydrazide beads were then denatured and reduced by 6 M guanidine and 5 mM DTT in 100 mM NaHCO_3_, pH 8.3, for 1 h at 37° C, and alkylated with 15 mM iodoacetamide for 1 h in the dark at RT. After 3 consecutive washes in 6 M guanidine followed by 3 additional washes in 100 mM NaHCO_3_, pH 8.3, the resin was resuspended as a 50% slurry in 100 mM NaHCO_3_, pH 8.3, and sequencing grade trypsin (Promega) was added at a 1:100 (w:w) trypsin-to-protein ratio. The sample was then digested on-resin overnight at 37° C. Nonspecifically bound tryptic peptides (non-glycopeptides) were removed by washing the resin three times with 6 M guanidine, five times with 100 mM NaHCO_3_, pH 8.3. The beads were then resuspended as 50% slurry in 100 mM NaHCO_3_ buffer pH 8.3, prepared in ^18^O water. Captured *N*-linked glycopeptides were released from the resin and labeled with ^18^O by incubation with Peptide-*N*-glycosidase F (PNGase F) (500,000 U/ml, glycerol free stock solution, New England Biolabs, Beverly, MA) at a ratio 1 μl of PNGase F per 400 μl of slurry (50% beads-50% NaHCO_3_ buffer in ^18^O water) overnight at 37° C. The released deglycosylated peptides labeled with ^18^O were collected from the supernatant and the beads were washed twice with ^18^O water. The supernatant and washes were combined, desalted using Hypersep C18 column (Thermo Scientific) according to manufacturer’s instructions, and lyophilized under vacuum.

#### LC-MS/MS analysis of enzymatically deglycosylated peptides

LC-MS/MS measurements were carried out on an Acquity UPLC M-class system coupled to a Thermo-Fisher Scientific Q Exactive (QE) hybrid quadrupole-orbitrap mass spectrometer. All separations were performed using 0.1% formic acid solution (v/v) in water as solvent A and 0.1% formic acid solution (v/v) in acetonitrile as solvent B. A 5 μL aliquot of desalted peptide resuspended in solvent A was loaded onto a C18 Trap column (Symmetry C18 Trap column 100Å, 5 μm, 180um x 20mm; Waters) and desalted further for 3 minutes at a flow rate of 10 μL per minute using solvent A. Next, peptide mixtures were fractionated on an analytical column (HSS T3 1.8 μm 75μm x 150mm; Waters). Separation was achieved by applying a linear gradient of 3–35% solvent B over 70 minutes at a flow rate of 450 nL per minute. The QE was operated using a data-dependent top10 method, which dynamically chooses the most abundant precursor ions from the survey scan (375 to 1500 m/z) for higher energy collisional dissociation (HCD) fragmentation. Parameters used for data-dependent acquisition were as follow: full MS scan range: 375 to 1500; MS/MS scan fixed first mass: 100; full MS scan AGC target: 3 × 10^+6^; MS/MS scan AGC target: 5 × 10^+4^; total cycle time: 1 s; dynamic exclusion duration: 15 s; spray voltage: 2.5 kV; capillary temperature: 300°C; S-lens RF level: 60 V. The parent ion resolution was 70,000 FWHM and the fragment ion resolution was 17,500 FWHM. The LTQ Velos ESI-positive ion calibration solution (Thermo-Fisher Scientific) was used to externally calibrate the instrument prior to sample analysis. Data were lock-mass corrected during the acquisition using background ions from polysiloxanes (*m/z* 371.1012 and 445.1200) to improve mass accuracy of precursor ions.

#### Data analysis

*De novo* and database-driven sequencing analyses were performed using De novo, PEAKS-DB, and SPIDER modules of the PEAKS Studio 8.5 software (Bioinformatics Solutions Inc., Waterloo, Canada). The raw data files were searched against a homemade database that combined 817 IgG sequences extracted from GenBank and the NCBI’s human reference proteome database (RefSeq release 11/28/2017). Database search and *de novo* sequencing were carried out using the following parameters: Carbamidomethylation of Cys (+57.02 Da), oxidation of Met (+15.99 Da), deamidation Asn and Gln in ^16^O water (+0.9840 Da), deamidation Asn and Gln in ^18^O water (+2.9883 Da), and C-terminal ^18^O_2_ labeling (+4.0084 Da) were set as variable modifications. Trypsin was selected as the digesting enzyme and up to three missed cleavage sites were allowed. Precursor and fragment error tolerances were adjusted to 10 ppm and 0.02 Da, respectively. *De novo* peptide sequences with an average local confidence score (ALC) of at least 70% were searched against the homemade antibody database, using the SPIDER module of PEAKS Studio software and NCBI/BLAST, to resolve some of the amino acid assignment ambiguities of the *de novo* sequencing. Peptides identified with a consensus NXS/T (with X not proline) motif and a modification at the asparagine due to incorporation of a single ^18^O isotope (a mass shift of +2.9883 Da at the site of modification) were regarded as potential *N*-linked glycopeptides. Those identified with a modification of deamidation at glutamine or at asparagines (a mass shift of +0.9840 or +2.9883 Da due to incorporation of a single ^16^O or ^18^O isotope, respectively) but not in the consensus sequence, were regarded as nonenzymatically deamidated peptides. Peptides showing significant similarity to the V-region of IgG molecules were searched against the IMGT germline V gene database (International ImMunoGeneTics information system, http://www.imgt.org) using NCBI/IGBLAST search tool. The top database sequence hit was used to map the pre-annotated FR/CDR boundary information to the query.

### Statistical analysis

Statistical analysis was performed using GraphPad Prism 7 (GraphPad Software, San Diego, CA). Two-sided Mann-Whitney test was used to explore the differences in glycosylation between AAV patients and healthy controls. For paired analyses, we used paired Student’s *t* test or Wilcoxon matched-pairs signed rank test as indicated in the legends. Bonferroni correction for multiple testing was performed throughout, with final significance thresholds depicted in the tables with results. Association of glycan traits with disease activity and severity were explored using Pearson correlation coefficients. Logistic regression was used to generate receiver-operating characteristic (ROC) curves and calculate the area under each ROC curve (AUC), with galactosylation-derived glycan trait as the predictor variable and one of two dichotomous outcomes: active AAV when compared with AAV patients in remission, or active AAV patients versus healthy controls. An optimal cut-off point for each analysis was defined using the Youden Index [47], which is the maximum of the sum of sensitivity and specificity. Positive likelihood ratios (LRs) for patients with active disease or when in remission or controls were calculated at these optimal cut-off points.

## Results

### Changes in Fc glycosylation profiles of total polyclonal IgG with disease activity

**Fig 2:**
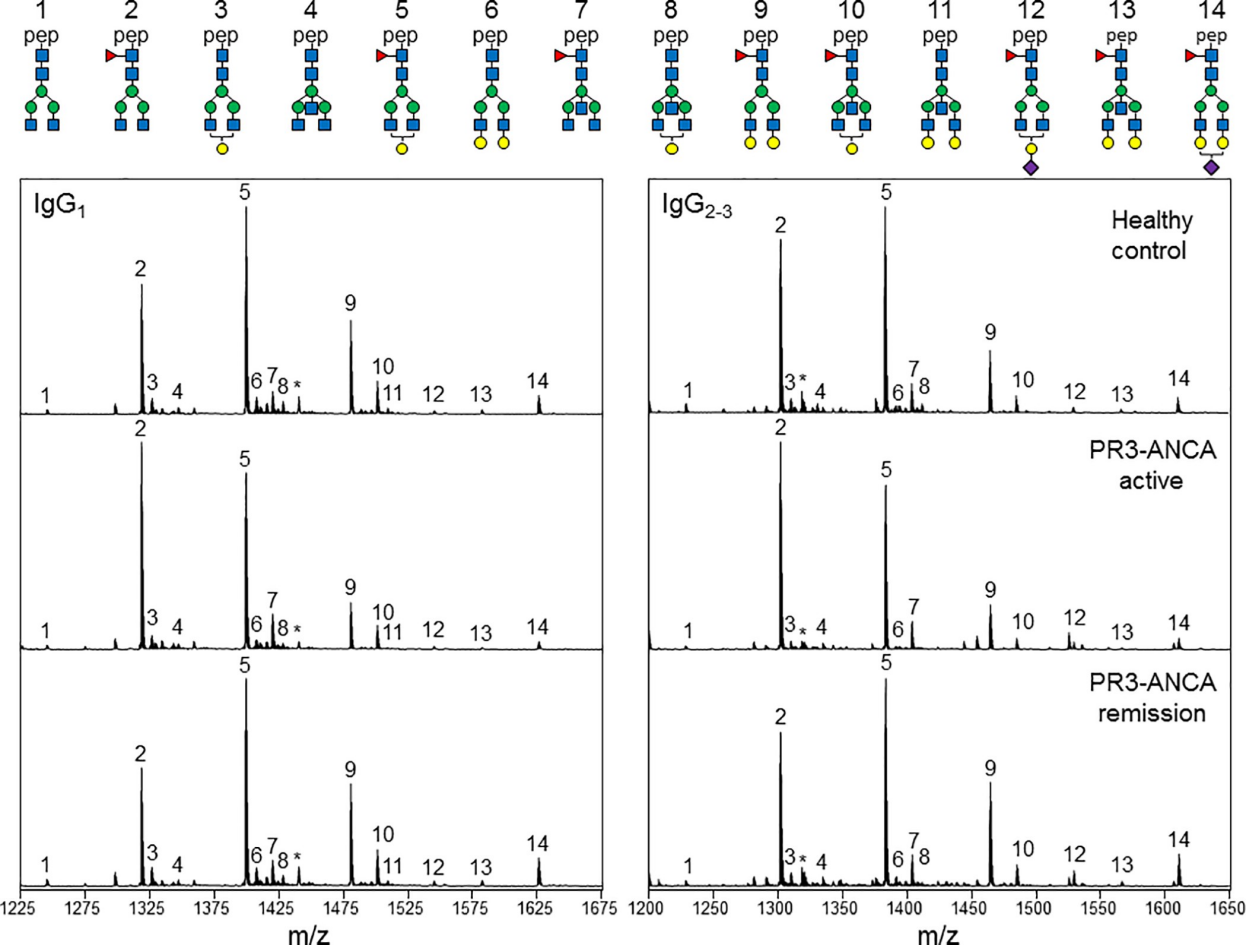
ESI-QTOF-MS spectra of total plasma IgG Fc *N*-glycopeptides. Representative LC-MS signals of the doubly protonated glycopeptide species are shown for a healthy control (top panels), a PR3-ANCA patient in active phase (middle panels), and the same patient in remission (bottom panels). Structures of the most abundant glycoforms detected in the present study are depicted above the upper panels with the numbers corresponding to the respective peaks on each MS spectra. Pep: peptide moiety, blue square: *N*-acetyl glucosamine, red triangle: fucose, green circle: mannose, yellow circle: galactose, purple diamond: *N*-acetyl neuraminic acid (sialic acid), *: contaminant or irrelevant peak.

Prior studies [36, 48] have shown that Ig Fc *N-*glycan profiles in patients with AAV when compared to healthy individuals are altered toward a more pro-inflammatory pattern. To replicate and extend these findings further, we analyzed the Fc-glycan profile of IgG isolated from serum or plasma samples of 30 patients with biopsy-proven AAV and 19 control subjects (characteristics provided in the supplementary material, S2 Table). IgG was digested by trypsin, and the resulting Fc glycopeptides were separated and detected using coupled liquid chromatographic electrospray mass spectrometry procedures (LC-ESI-MS). Fig 2 shows representative examples of the IgG Fc glycosylation profiles obtained for a PR3-ANCA patient in active phase and in remission, and the corresponding age- and sex-matched healthy control. Compared to healthy controls, the PR3-ANCA active sample contained the highest levels of agalactosylated structures G_0_F (peak 2), whereas levels of digalactosylated structures G_2_F (peak 9) and terminally sialylated glycoforms G_1_FS and G_2_FS (peak 12 & 14, respectively) were lowest. In contrast, the glycan profile of the same patient in remission most resembled that of healthy controls.

**Fig 3:**
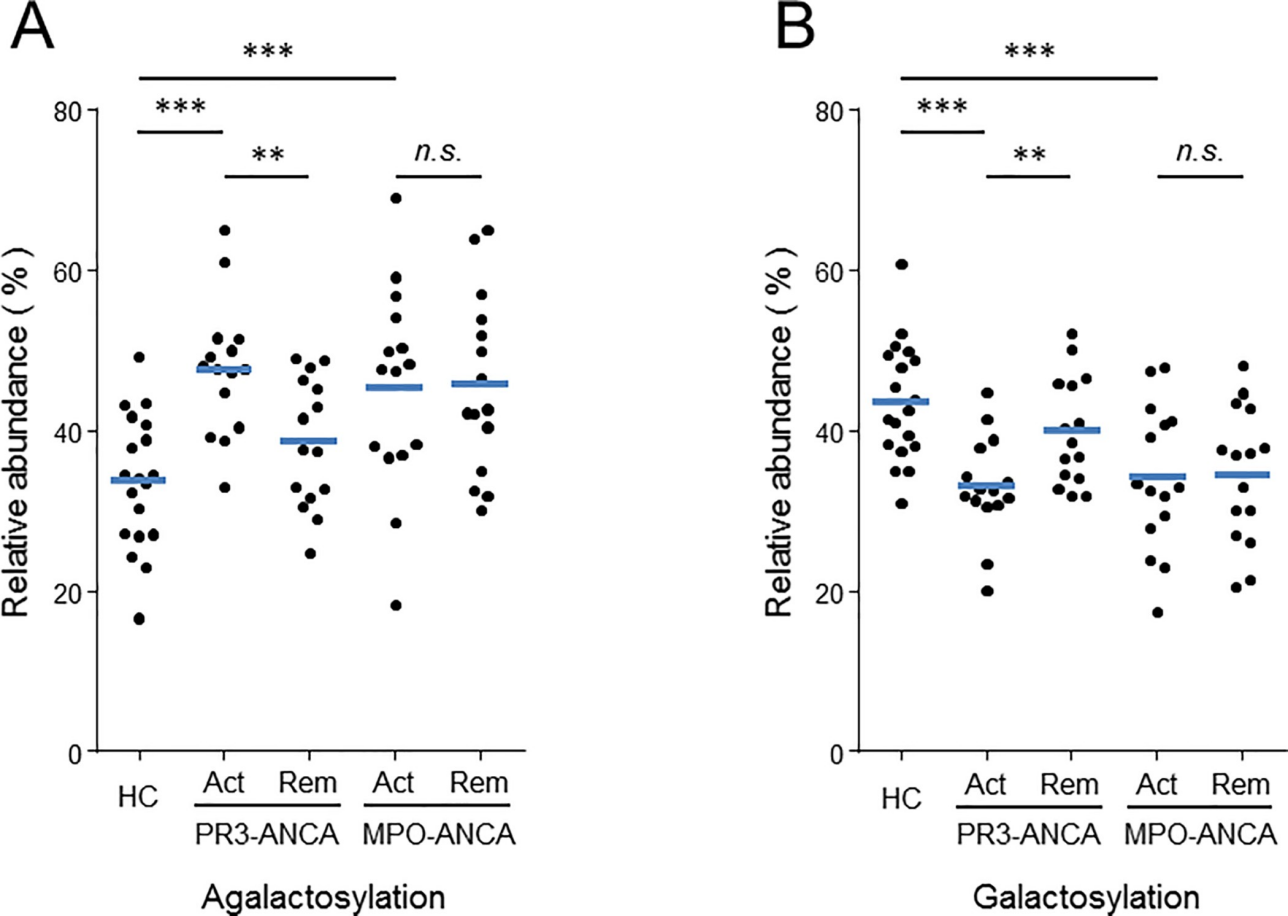
Changes in IgG Fc glycosylation with disease activity; comparison of the IgG_1_ Fc glycosylation features of ANCA patients and controls. A: agalactose-derived glycan traits. B: galactose-derived glycan traits. Each glycosylation feature is expressed as a percentage of the total ion abundance of the 14 glycoforms analyzed in this study. HC: Healthy control, Act: Active phase, Rem: Remission. Median values are indicated by blue horizontal bars. Significant differences are indicate by two asterisks (*p* < 0.01) or three asterisks (*p* < 0.001). n.s.; non significant.

Fourteen *N*-glycopeptides released from either IgG_1_ or IgG_2/3_ Fc CH2 domains were detected in quantifiable amounts in AAV patients and control samples. Individual values for relative abundance of fourteen IgG_1_ Fc *N*-glycan peaks and the associated glycan traits are shown in S3 and S4 Tables. Pairwise multiple comparison tests were used to determine if substantial quantitative differences exist in the relative abundance of individual *N*-glycan peaks among the sample groups (S3 Table). Pairwise comparison between individual *N*-glycan peaks for the control samples and for samples from patients with PR3-ANCA and MPO-ANCA revealed several quantitative, statistically significant differences (*p-*values < 0.05) highlighted in bold. These included G_0_F, G_2_F, and G_o_FN structures that were found to undergo significant changes in relative abundance both in the serum of patient with PR3-ANCA and MPO-ANCA compared with healthy controls. The variation in Fc glycosylation profiles are more readily appreciated from the changes in abundance of galactose-derived glycan traits (Fig 3). Specifically, the normalized intensities of the agalactosylated IgG_1_ Fc species were significantly higher in patients with active disease than controls (Fig 3A). Conversely, the overall IgG_1_ Fc galactosylation (Fig 3B) was significantly reduced in patients with active disease compared to controls. Our data also indicate significant differences between MPO- and PR3-ANCA diseases regarding the association of aberrantly glycosylated IgG levels with disease activity. Compared to active disease, agalactosylation in neutral glycan species was lowest, and not significantly different from controls, in the PR3-ANCA patient group in remission (Fig 3A). In contrast, Fc *N*-glycans levels of patients with MPO-ANCA did not correlate with disease activity. Notably, no statistically significant difference was found in the degree of galactosylation of the IgG_1_ Fc glycoforms at the time of active disease and remission (Fig 3A and S4 Table). Samples from patients with MPO-ANCA continued to exhibit a deficit of terminal galactose residues during disease remission compared to healthy controls.

Two-tailed Pearson correlation analysis revealed a strong correlation between IgG_1_ and IgG_2/3_ galactosylation levels (S1 Fig and S5 Table), indicating that aberrant agalactosylation of Fc *N*-glycans is likely to be a common event in all IgG subclasses. Regarding sialylation, the relative abundance of terminally sialylated glycoforms G1FS (Fig 2, peak 12) and G2FS (Fig 2, peak 14) were equivalently reduced in both PR3- and MPO-patients with active disease compared to healthy controls. Two-tailed Pearson correlation analysis showed a strong correlation between sialylation of the IgG_1_ and IgG_2/3_ Fc *N*-glycans and the level of galactosylation in AAV patients and healthy controls (Supplemental S6 Table and S1B and S1C Fig). No significant differences were found in the relative abundance of fucosylated and bisected structures in AAV patients compared to healthy controls (Supplemental S4 Table). Each Fc *N*-glycan trait was statistically examined for evidence of correlation with disease activity and severity expressed in term of BVAS score (Table 1). The incidence of Fc galactose-derived glycan trait was inversely correlated with the BVAS in a weak but statistically significant fashion in PR3-ANCA (r = -0.39, p = 0.029) but not MPO-ANCA samples. There was no significant correlation between disease activity and levels of Fc sialylation, fucosylation, and bisection in either PR3- or MPO-ANCA samples.

**Table 1:** Correlation analysis between IgG_1_ Fc glycan traits and BVAS ^a^.

Disease	Glycosylation traits	Pearson r ^b^	*p* value ^c^
PR3-ANCA	Galactosylation	-0.39	**0.029**
	Sialylation	-0.13	0.473
	Fucosylation	0.4	0.234
	Bisection	-0.31	0.088
MPO-ANCA	Galactosylation	0.14	0.499
	Sialylation	0.36	0.066
	Fucosylation	0.32	0.101
	Bisection	-0.19	0.349

^**a**^ BVAS, Birmingham Vasculitis Activity Score, is a composite score expressing organ involvement; the higher the global score achieved, the more severe the disease.

^**b**^ Two-tailed Pearson correlation analysis.

^**c**^ *p* values < 0.05 are highlighted in bold and considered significant.

The ability of galactose-derived glycan trait to distinguish patients with active disease from those in remission or from healthy controls was quantified by measuring the area under each receiver-operator curve (AUC) and calculating positive likelihood ratios (LRs) (Supplemental S7 Table). AUC values obtained for galactose-derived glycan trait indicated significant ability of this marker to separate active PR3-ANCA samples from remission and from healthy control samples (range 0.78–0.86). The marker also showed a strong ability to separate active MPO-ANCA from healthy controls (AUC = 0.80), but no ability to distinguish patients with MPO-ANCA when their disease was active or when they were in remission (AUC = 0.51).

### Fc-glycosylation profile of anti-MPO-specific IgG

**Fig 4:**
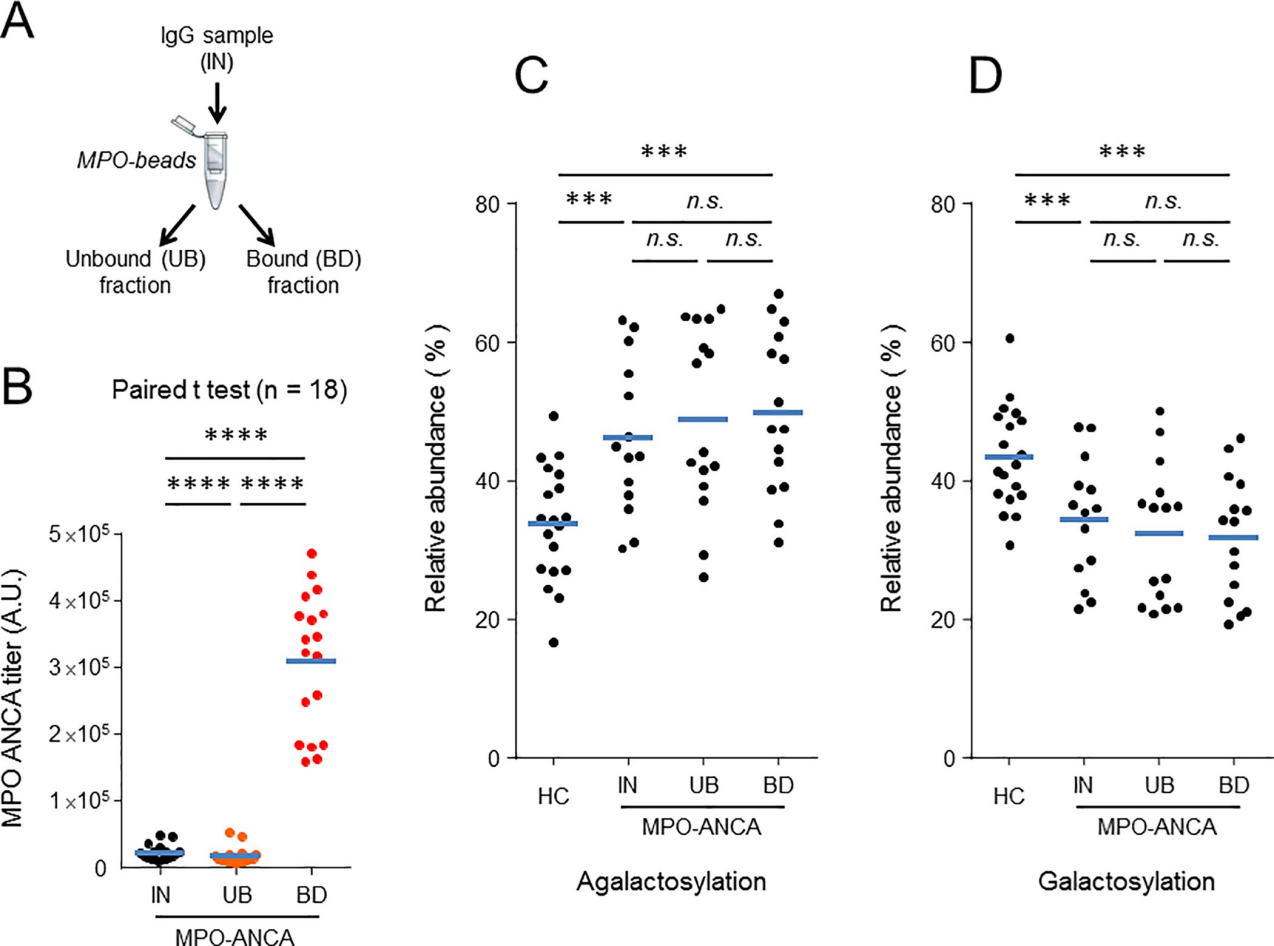
Fc-glycosylation features of anti-MPO enriched IgGs relative to unfractionated material and to healthy controls. A: Scheme of the affinity pull-down assay. IN: sample before fractionation, UB: unbound fraction (anti-MPO depleted IgG), BD: bound fraction (anti-MPO enriched IgG). B: Determination of MPO-ANCA titers of IgG pull-down fractions by ELISA. C and D: Comparison of glycosylsation features of IgG pull-down fractions and controls. HC: Healthy control. Median values are indicated by blue horizontal bars. Statistical differences were analyzed using a paired Student’s *t* test. ****: p < 0.0001, ***: p < 0.001, n.s.: non-significant.

Next, we examined whether MPO-ANCA-specific autoantibodies exhibit specific Fc glycosylation profiles that would help elucidate their role in disease pathogenesis. We were unfortunately unable to perform similar experiments for PR3-ANCA-specific autoantibodies because of the prohibitive cost of immunopurifying these autoantibodies. Affinity purification of anti-MPO autoantibodies was performed using IgG isolated from plasmapheresis fluids and antigen-coated beads, as outlined in Fig 4A. Characteristics of the MPO-ANCA positive patients from which plasmapheresis fluids were collected are presented in supplemental S8 Table. MPO-ANCA titers of bound (BD) and unbound (UB) fractions, and the sample before fractionation (IN), were evaluated by antigen-specific ELISA and normalized to the IgG concentration in each fraction.

The results presented in Fig 4B show significantly higher MPO-ANCA titers in BD fractions, and significantly lower titers in UB fractions compared to IgG samples before fractionation. Fc glycosylation profiles of affinity pull-down fractions were determined by LC-MS, and levels of glycopeptides were calculated after signal extraction and total ion abundance normalization. No significant differences in relative abundance of agalactosylated species (Fig 4C), overall galactosylation (Fig 4D), sialylation, bisected species, or fucosylation (not shown) were observed in any of the fractions.

### Anti-MPO-specific IgGs are hyperglycosylated in the fab region

**Fig 5:**
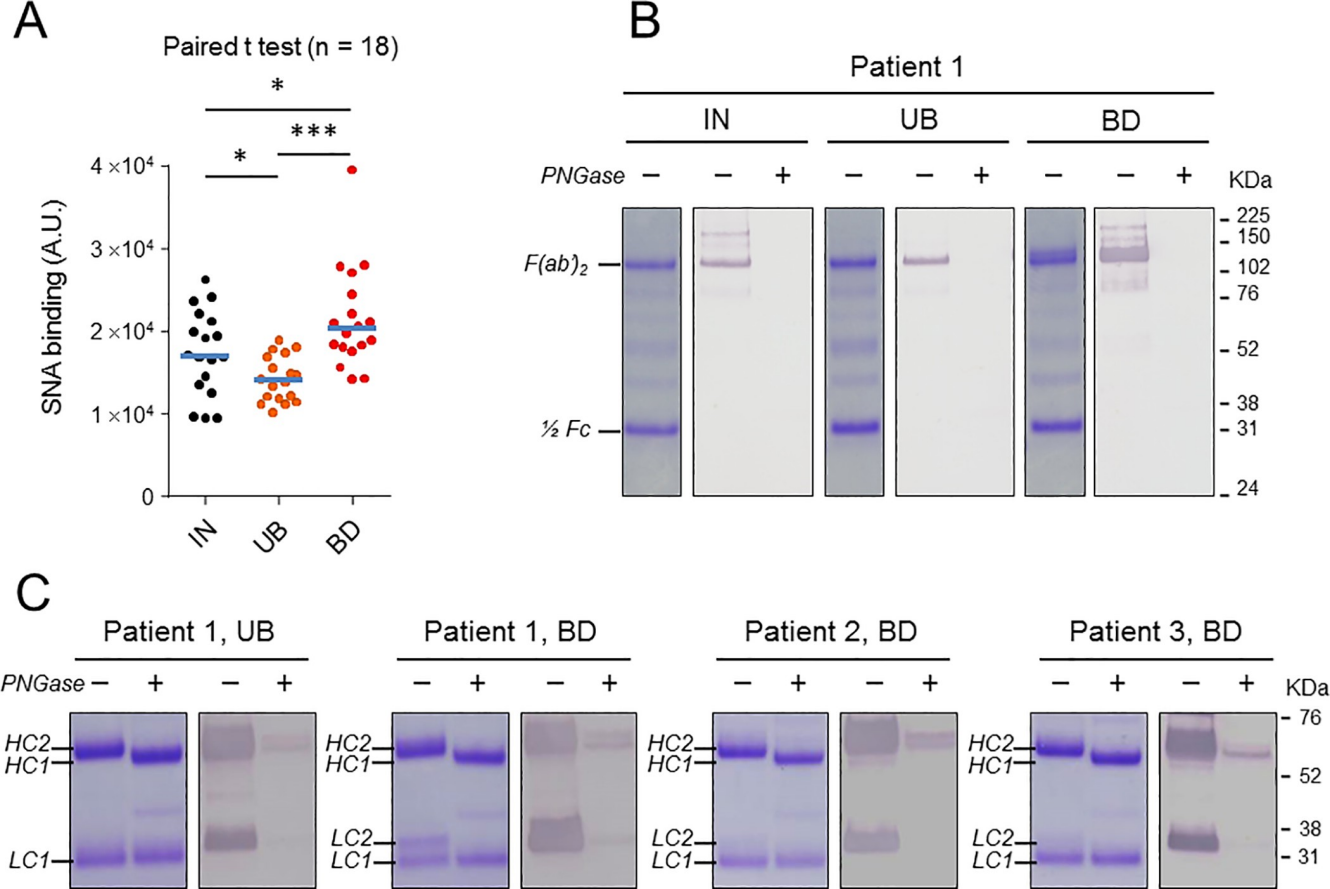
Sialic acid content of anti-MPO enriched IgGs relative to unfractionated material. Scheme of the affinity pull-down assay as in Fig 4. IN: sample before fractionation, UB: unbound fraction (anti-MPO depleted IgG), BD: bound fraction (anti-MPO enriched IgG). A: Determination of sialic content of IgG pull-down fractions by enzyme-linked lectin assay (ELLA). Median values are indicated by blue horizontal bars. Statistical differences were analyzed using a paired Student’s *t* test. ***: p < 0.001, *: p < 0.1. B: Digestion of pull-down fractions with IdeS protease produces F(ab’)_2_ and Fc fragments that were separated by SDS-PAGE under non-reducing conditions and detected by Coomassie blue staining. Sialic acid content of IdeS digestion products was evaluated by lectin blot analyses. C: Incubation of IgG pull-down fractions with reducing agent DTT results in dissociation of heavy and light chains that were separated by SDS-PAGE, detected by Coomassie blue staining, and evaluated for sialic content by lectin blots. LC: light chain, HC: heavy chain. Note the double band in Patient 1, BD sample, of which mainly the upper one (LC2 band) is visualized by SNA lectin. The two other samples (Patients 2 and 3) exhibited only one light chain and one heavy chain band following Coomassie blue staining. All samples in panels B and C were treated with (+) or without (-) PNGase-F prior to separation on SDS-PAGE. Removal of *N-*glycans by PNGase F treatment reduced SNA binding.

Glycosylation on MPO-ANCA Fab fragments is elevated during active disease [49]. To confirm and investigate this phenomenon further, the degree of sialylation of IgG pull-down fractions was determined by an enzyme lectin assay using *Sambucus nigra* agglutinin (SNA), which specifically binds the terminal sialic acid. The anti-MPO enriched (BD) and anti-MPO depleted (UB) materials, and the purified IgG sample before fractionation (IN) were analyzed. The results presented in Fig 5A are normalized to the IgG concentration in each fraction and show a significantly higher binding of the biotinylated SNA to BD fractions, and lower levels of binding to UB fractions, compared to IgG samples before fractionation. No significant correlation was found between SNA binding levels to each fraction and the BVAS score (S9 Table). To determine the binding site of the lectin, we measured the SNA binding to F(ab’)_2_ and Fc fragments by lectin blot-based analysis. F(ab’)_2_ and Fc fragments were prepared by enzymatic digestion of IgG pull-down fractions with recombinant *Streptococcus pyogenes* IdeS, a protease that cleaves all human IgG subclasses within the lower hinge region [50]. The results presented in Fig 5B indicated that SNA bound exclusively to F(ab’)_2_ fragment, with no detectable binding to Fc fragments. PNGase F treatment completely abolished SNA binding confirming that sialic addition to F(ab’)_2_ fragments occurred on *N*-glycans. In BD fractions, there was an obvious increase in SNA binding to F(ab’)_2_. The results of SDS PAGE and lectin-blot analyses following reduction of IgG interchain disulfide bonds by dithiothreitol (DTT) are shown in Fig 5C. Both the BD and UB fractions gave the expected two bands for the heavy and light chains on reducing SDS-PAGE (HC2 and LC1 bands) as well as a size shift of about 3 KDa of the heavy chain (from HC2 to HC1 positions) visible on Coomassie blue-stained gels after PNGase F treatment, corresponding to loss of Fc-linked *N*-glycans. Surprisingly, the BD fraction of some patients exhibited an additional band detectable by Coomassie blue staining above the light chain (LC2 band, patient 1). This additional band was completely abolished by PNGase treatment, suggesting that it is due to additional *N*-glycans in the Fab-portion of the light chain. The additional LC2 electrophoretic band was observed in only two of the eighteen patient samples investigated. Lectin blot with SNA gave strong staining of one or more overlapping HC and LC bands of increased molecular weight moving respectively around HC2 and LC2 position, in line with numerous reports indicating that carbohydrate addition to Fab may occur on the heavy and light chain of IgG molecules. PNGase treatment completely abolished SNA binding to LC2 bands and significantly reduced its binding to HC2 bands, but also permits clear resolution of a doublet of heavy chains moving around HC2 position. The band of higher molecular weight in the doublet was more prominent in the anti-MPO enriched (BD) material in comparison to unbound (UB) fraction (Fig 5C, patient 1). This was observed for most samples subjected to SNA-lectin blot analysis (4/5) and indicated that, relative to non-ANCA-specific IgG (i.e. MPO ANCA depleted IgG isolated from the same patient), a minor but significant fraction of anti-MPO IgG molecules contained additional *N*-linked sialylated glycans in the Fab-portion of their heavy chains.

### Carbohydrate profile of *N*-glycans released from IgG heavy and light chains

**Fig 6:**
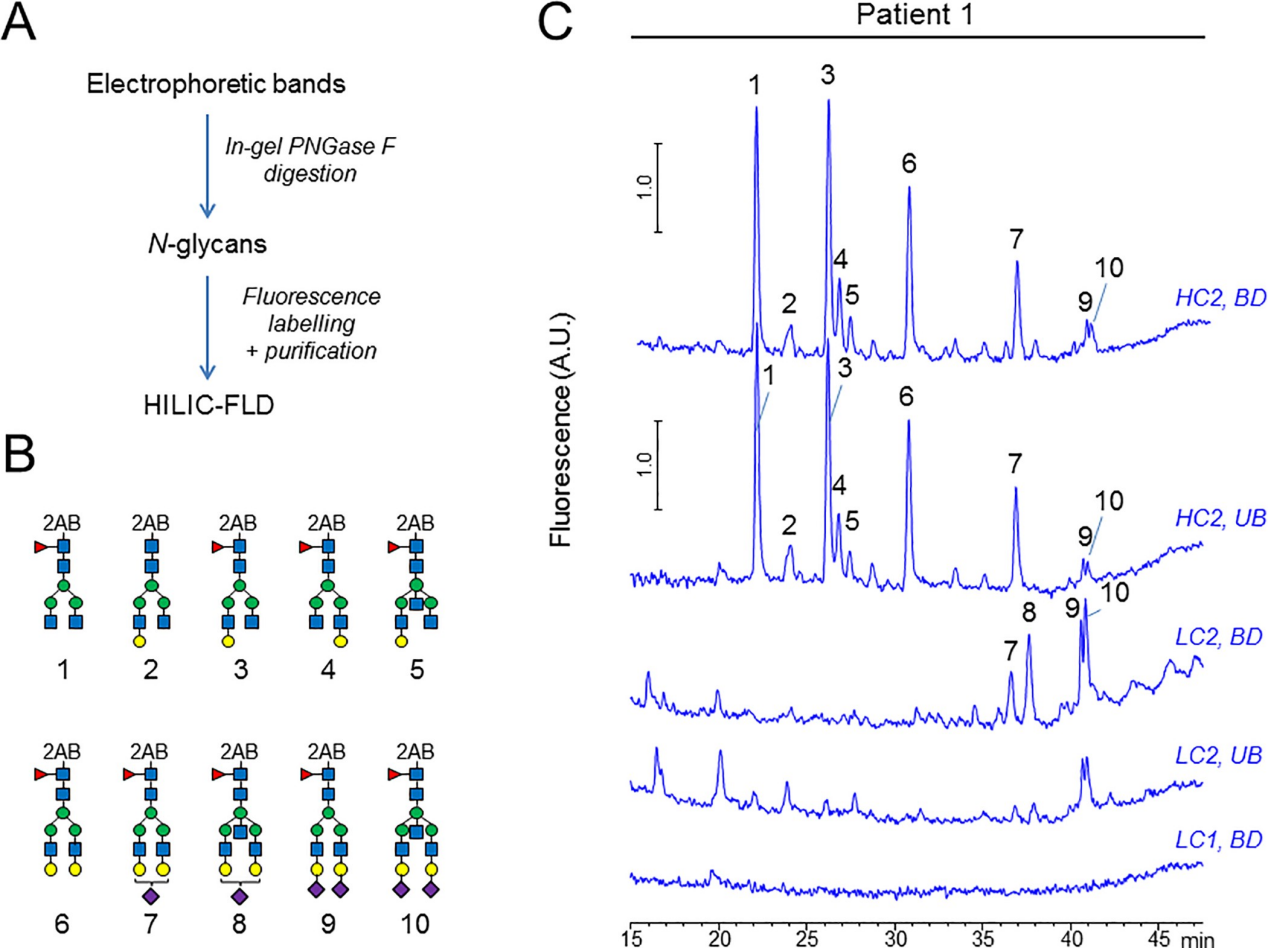
Carbohydrate profile of anti-MPO-enriched IgGs relative to unbound fraction. A: Glycans were released by in-gel PNGase F treatment and labeled with 2-aminobenzamide. The labeled glycans were separated by HPLC with fluorescence detection. B: Structures of the most abundant 2-AB labeled glycans detected with numbers corresponding to the respective peaks on each chromatogram. C: Glycosylation profiles obtained for the anti-MPO enriched (BD) and anti-MPO depleted (UB) materials. 2AB: 2-aminobenzamide, blue square: *N*-acetyl glucosamine, red triangle: fucose, green circle: mannose, yellow circle: galactose, purple diamond: *N*-acetyl neuraminic acid (sialic acid), LC: light chain, HC: heavy chain, UB: unbound fraction (anti-MPO depleted IgG), BD: bound fraction (anti-MPO enriched IgG), HILIC: Hydrophilic interaction chromatography.

To further validate these findings, electrophoretic bands of anti-MPO enriched (BD) and depleted (UB) materials were excised, *N*-glycans were released by PNGase F in-gel digestion, and labeled with 2-aminobenzamide (2-AB). The 2-AB labeled glycans were then separated and characterized by hydrophilic interaction chromatography with fluorescence detection (HILIC-FLD). The glycosylation profiles of the HC bands obtained from BD and UB samples were very similar and typical of Fc-linked *N*-glycans (Fig 6C, HC2 traces). In contrast, marked differences in glycosylation were observed for the LC bands. The additional LC2 electrophoretic band detected in BD fraction showed the presence of Fab-linked *N*-glycans, and a higher level of glycosylation than in the corresponding band in the UB fraction (Fig 6C, LC2 traces). In contrast, no glycans were found on the LC1 band (consistent with the lack of staining of this band by SNA).

### Enrichment of anti-MPO-specific IgG by lectin fractionation

**Fig 7:**
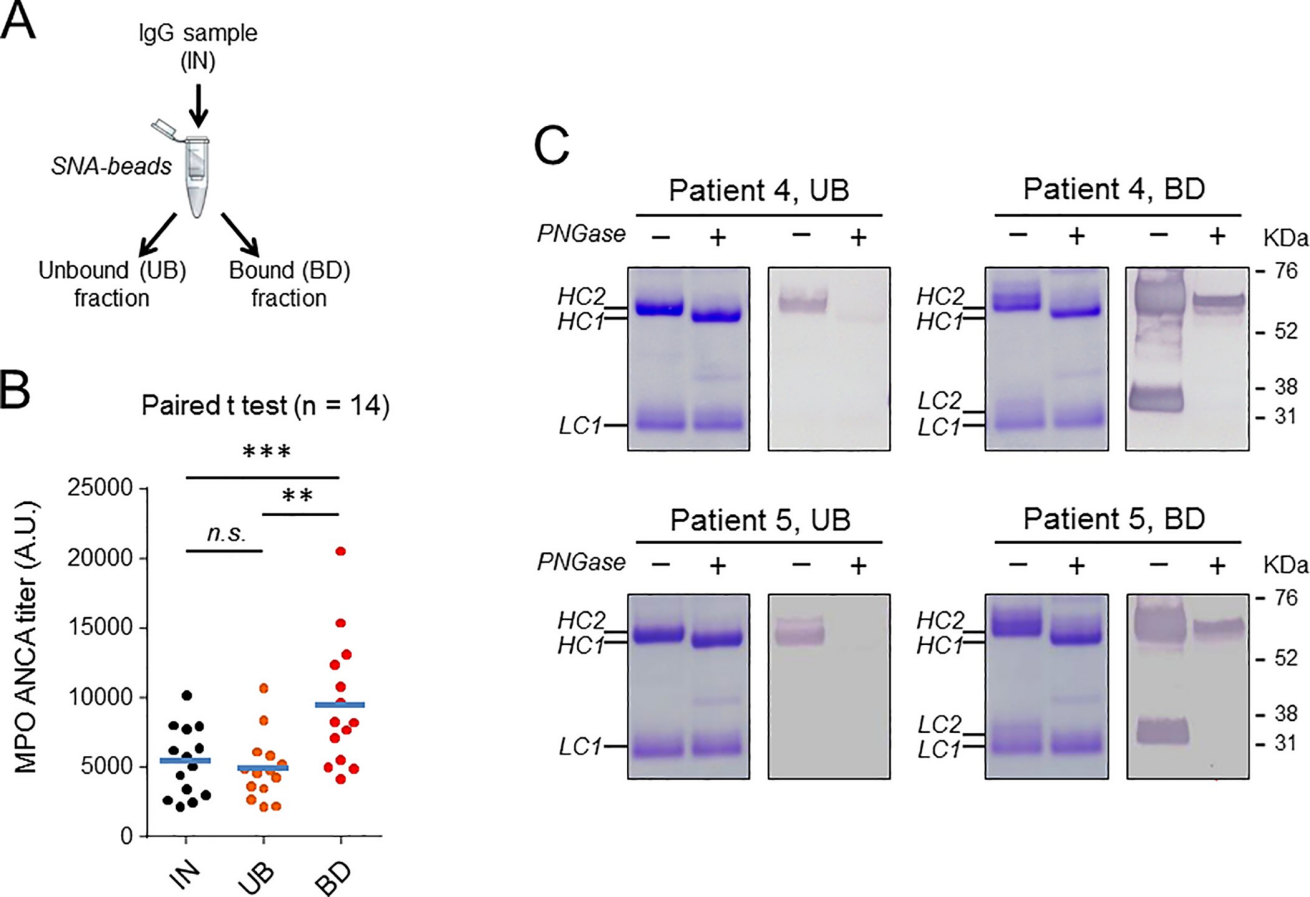
Anti-MPO titers and sialic acid content of SNA-enriched IgGs relative to unbound fraction. A: Scheme of the affinity pull-down assay. IN: sample before fractionation, UB: unbound fraction (Sialic-depleted IgG), BD: bound fraction (Sialic-enriched IgG). B: Determination of MPO-ANCA titers of IgG pull-down fractions by ELISA. Median values are indicated by blue horizontal bars. Statistical differences were analyzed using a paired Student’s *t* test. ***: p < 0.001, **: p < 0.01, *n*.*s*., not significant. C: Separation of heavy and light chains of IgG pull-down fractions by SDS-PAGE under reducing condition. The protein bands were stained with Coomassie blue and their Sialic acid content was evaluated by lectin blot analyses. Samples in panel C were treated with (+) or without (-) PNGase-F prior to separation on SDS-PAGE. LC: light chain, HC: heavy chain, UB: unbound fraction (Sialic-depleted IgG), BD: bound fraction (Sialic-enriched IgG).

To confirm anti-MPO-specific IgG hyperglycosylation in the Fab region, IgG isolated from plasmapheresis fluid from patients with MPO-ANCA was enriched or depleted for sialylated IgG using SNA lectin fractionation, as outlined in Fig 7A. MPO-ANCA titers of collected fractions corresponding to bound (BD) and unbound (UB) materials were evaluated by ELISA. The results presented in Fig 7B are normalized to the IgG concentration in each fraction and show significantly higher MPO-ANCA titers in BD fraction, compared to UB fraction or to IgG sample before fractionation. This was observed in the majority of patient samples investigated (total number of patients tested = 14) and indicated that anti-MPO-specific IgG were enriched by the lectin affinity pull-down assay. Next, sialylation levels of IgG pull-down fractions were evaluated by SDS-PAGE followed by lectin blotting with SNA. Representative SDS-PAGE and lectin blots are presented in Fig 7C (patient 4 and 5). A strong enrichment of sialylated IgG was observed in BD fractions, compared to UB fractions. In all preparations, treatment with PNGase F effectively reduced SNA binding. Interestingly, additional bands detectable by Coomassie blue staining above the LC1 and HC2 bands were observed in BD fractions of all donor samples after SNA lectin fractionation (HC3 and LC2 bands in Fig 7C). These high molecular weight bands were significantly reduced or completely abolished by PNGase treatment, suggesting that they are due to additional *N*-glycans in the Fab-portion.

### Carbohydrate profile of IgG glycovariants separated by lectin chromatography

**Fig 8:**
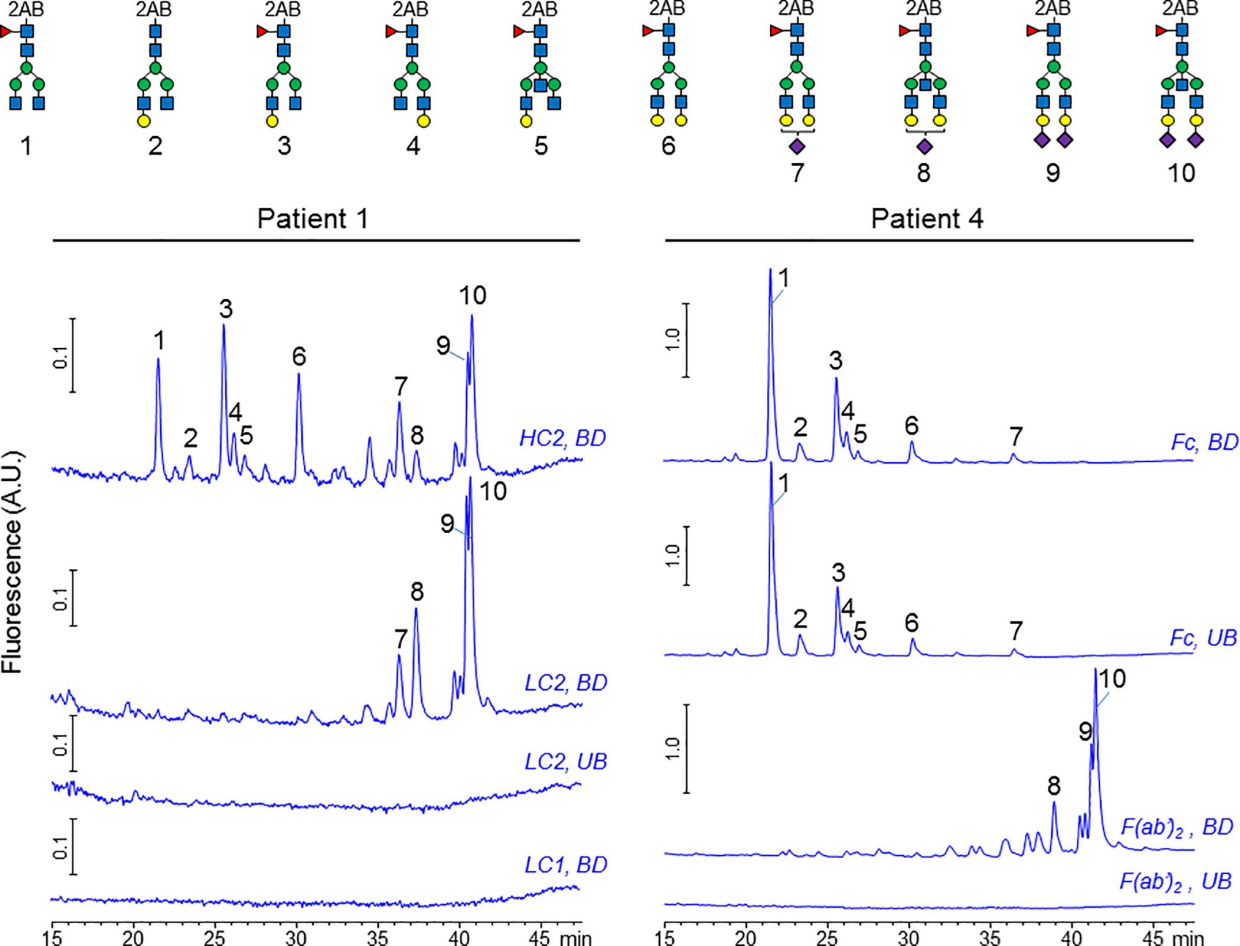
Carbohydrate profile of SNA-enriched IgGs relative to unbound fraction. Glycans were released by in-gel PNGase F treatment and labeled with 2-aminobenzamide. The label glycans were separated by HPLC with fluorescence detection. Top: Structures of the 2-AB labeled glycans detected with numbers corresponding to the respective peaks on each chromatogram. Bottom: HPLC chromatograms of 2AB-labeled *N*-linked glycans. LC: light chain, HC: heavy chain, UB: unbound fraction (Sialic-depleted IgG), BD: bound fraction (Sialic-enriched IgG).

Carbohydrate profiling by HPLC of *N*-glycans released from IgG HC and LC chains (Fig 8, patient 1), and from Fc and F(ab’)_2_ fragments (Fig 8, patient 4), further substantiate these findings. Glycans released from additional HC bands displayed a mixture of Fc-linked and Fab-linked glycans (HC2, BD). In contrast, the additional LC electrophoretic band showed only Fab-linked glycans (LC2, BD). No glycans were found on LC1 band or on LC2 band in BD fraction. We found a substantial enrichment of terminally sialylated glycoforms in BD fractions when F(ab’)_2_ fragments were used as the starting material (Fig 8, F(ab’)_2_ traces), but no increase in Fc-sialylation was detected in BD fraction, compared to UB fraction (Fc traces). These results strongly suggest that enrichment capability of SNA for anti-MPO IgG is mainly driven by interactions of the lectin with hypersialylated Fab-portion of the autoantibodies.

### Mapping *N*-linked glycosylation sites on fab fragment

We determined the exact localization and abundance of *N*-glycans on purified IgGs isolated from three patients with MPO-ANCA. LC-MS/MS measurements of the three IgG solutions yielded a combined compound list of 24678 MS/MS scans. A list of 7764 peptides, representing 2425 unique peptide sequences, was generated from *de novo* sequencing of these MS signals. The identified sequences were examined for the presence of the known consensus *N*-linked glycosylation motif (NXT/S, where X is any amino acid except proline). This analysis showed that 94% of identified peptides contained the motif. The procedure reliably identified the conserved Fc glycosites at position 297 of the CH2-domain, thereby validating the strategy for identification of *N-*glycosylation sites (not shown). It also allowed identification of an additional 108 peptides having *N*-glycosylation sites on Fab fragments (S10 Table). Sequence similarity search against the IMGT human germline IG genes [51] indicated that the majority of captured glycopeptides from Fab fragments included *N-*glycosylation sites not encoded in the germline, an indication that they have been acquired by somatic hypermutation. The new *N*-glycosylation sites were found mainly in framework region 3 (FR3) that encodes structural integrity of the immunoglobulins (S10 Table). A smaller fraction of the NXT/S motif was present in the complementary determining regions (CDRs) that contact antigens.

## Discussion

The present study uses multiple approaches to examine changes in IgG Fc and Fab glycosylation with disease activity in patients with AAV. Glycosylation analyses of IgG from AAV patients have been performed before primarily on total IgG derived from peripheral blood [36, 48] or on anti-PR3 specific IgG [44, 52]. The only previous investigation on the glycosylation states of anti-MPO specific IgG was performed using lectin-binding assays [49]. The glycosylation state of anti-MPO-specific autoantibodies has not otherwise been investigated.

Early studies have shown that, as in other autoimmune diseases, PR3-ANCA and MPO-ANCA are associated with alterations in the glycosylation of IgG including a decrease in sialylated and galactosylated structures [36, 48]. Intriguingly, this aberrant glycosylation was found to be limited to the Fc, but not the Fab portion, suggesting that there was no defect in the general glycosylation or processing machinery of IgG-secreting plasma cells [36]. A subsequent report by Espy *et al* [52] indicated that the development of vasculitis in patients with GPA could be linked with a low sialylation levels of IgG and, in particular, anti-PR3 antibodies. In that study, enzymatic desialylation of IgG from patients with GPA in remission was found to significantly increase neutrophil superoxide generation [52]. There was a negative correlation between disease activity and anti-PR3 IgG sialylation levels [52]. However, the negative correlation between BVAS and anti-PR3 IgG sialylation was not confirmed in a more recent report by Wuhrer et al [44] using high resolution mass spectrometry to analyze the Fc fragment glycosylation (and sialylation). This discrepancy may be related to differences in the methods used. The initial study [52] used SNA lectin and a lectin-binding assay to evaluate the degree of sialylation of IgG from patients. Until recently, it was thought that SNA interacts mainly with Fc-bound sialic acid. Later studies, including data presented herein, indicate that this lectin binds preferentially to sialic residues in the Fab region of IgG and the binding may not reflect Fc glycosylation [53–55]. Thus, it was important to use analytical strategies that differentiate between Fc and Fab-linked glycans to examine the association of glycosylation aberrancies in ANCA vasculitis with disease activity.

We first analyzed IgG Fc glycosylation of total serum IgG from patients by liquid chromatography electrospray mass spectrometry. This highly sensitive method of IgG Fc glycan profiling is not confounded by other glycans that might be linked to the IgG molecule, such as Fab-linked glycans [56, 57]. Consistent with previous reports [36, 44, 48] [52], we found that the Fc domain of IgG_1_ from AAV patients were hypogalactosylated and hyposialylated compared to heathy controls. We also found a correlation between Fc galactosylation and sialylation for total IgG_1_ of both healthy controls and AAV patients. This is in line with previous reports documenting elevated levels of desialylated IgG that correlate with a deficit of galactose in different inflammatory diseases [58], and fits with the common contention that the reduced sialylation of Fc *N*-glycans in patients with active disease primarily reflects a lack of galactose rather than defective terminal sialylation. IgG Fc glycosylation profiles of IgG_2/3_ subclasses were similar and consistent with those observed for IgG_1_, indicating that alteration of IgG Fc glycosylation is most likely a common event in all IgG subclasses, perhaps as a result of an identical stimulation of IgG-secreting plasma-cells. The findings of altered IgG Fc galactosylation is not specific to AAV and has been described in other autoimmune or inflammatory diseases [59–61], cancer [62–65], and increased age [66–68].

Our analysis of IgG Fc glycosylation profiles of total serum IgG indicates that significant differences exist between MPO- and PR3-ANCA serotypes regarding changes in the level of galactosylated structures and terminally sialylated glycoforms. In PR3-ANCA patients, deficits of total IgG Fc galactosylation and sialylation correlate strongly with disease activity, and revert to normal levels with disease remission. In contrast, Fc *N*-glycans levels of patients with MPO-ANCA did not correlate with disease activity. Notably, patients with MPO-ANCA continued to exhibit a deficit of terminal sialic acid and galactose residues during disease remission compared to healthy controls. The observation that glycan traits normalize with remission in PR3-ANCA, but not in MPO-ANCA disease, is intriguing considering the fact that patients with PR3-ANCA have a significantly greater rate of relapse than patients with MPO-ANCA disease [10, 14–17]. This finding may imply a more prominent role for changes in IgG Fc glycosylation in the pathogenesis of PR3-ANCA than MPO-ANCA disease. It also raises the possibility that the humoral dysregulation driving the production of aberrantly glycosylated IgG may predate the clinical manifestations of the disease in MPO-ANCA disease, while the dysregulation may occur closer to the onset of clinical symptoms in PR3-ANCA disease. More detailed information on this question could be obtained from studying events very early in the process of disease development. Currently, however, no information is available on change of IgG Fc glycosylation in AAV patients before disease onset. Our analysis indicates that levels of IgG Fc galactosylation separates active PR3-ANCA from remission and from healthy controls suggesting that this glycan trait could be used as a marker of disease activity. Further analysis of serial samples during remission and preceding or at relapse is necessary to determine whether IgG Fc glycosylation could be used as a marker of risk or predictor of subsequent relapse.

In patients with MPO-ANCA, we found no significant differences between antigen-specific and total IgG with regards to relative abundance of Fc agalactosylated species, Fc sialylation, bisected species, and fucosylation. This is in marked contrast to the proinflammatory Fc glycosylation profiles found in anti-PR3 IgG [44]. The reason why anti-MPO specific IgGs analyzed here did not exhibit comparable Fc glycosylation changes that are more inflammatory in nature is unclear. Conceivably, this could be a consequence of differences in tissues/compartments in which the anti-MPO and anti-PR3 autoantibodies are produced, and the impact of local cytokines that regulate glycan processing on plasma cells present in these tissues/compartments. Local activation or control of the immune response within affected tissue has been described for several chronic inflammatory conditions [69]. Local ANCA production is consistent with the histologic presence of germinal centers in granulomas of ANCA patients [70, 71]. However, whether newly generated plasma cells derived from these germinal centers differentially process Fc-linked glycans remains to be established and warrants further investigations.

Our findings differ from those previously reported [49] that glycosylation on MPO-ANCA Fab fragment was elevated during active disease. That study used a lectin-binding assay and SNA lectin to evaluate the degree of sialylation of IgG from patients, thus providing limited insights into the molecular nature of Fab glycosylation. In our study, we have used a HPLC-based method with fluorescence detection to more fully characterize the glycans on Fc and Fab domains of anti-MPO antibodies and compared the glycosylation profiles with those of anti-MPO depleted IgG. The analysis of Fab glycosylation profiles revealed marked differences between anti-MPO IgG and IgGs depleted of ANCA. Levels of galactosylation and sialylation were higher in the Fab portion of anti-MPO antibodies. In contrast, and in agreement with our IgG Fc analyses by LC-ESI-MS, no significant differences in the Fc glycosylation profiles were detected between the anti-MPO enriched and anti-MPO depleted materials. Despite the fact that Fab glycosylation was higher in the anti-MPO enriched materials, they were not associated with disease activity

Interestingly, anti-MPO IgG of some patients exhibited additional electrophoretic bands of higher molecular weight with significantly higher levels of glycosylation than in corresponding bands of anti-MPO-depleted IgG. Using hydrazide glycoprotein capture and LC-MS/MS analysis, we identified 108 unique *N*-glycosylated peptides on Fab fragments. A sequence similarity search against the IMGT human germline IG genes demonstrated that the majority of glycopeptides from Fab fragments included *N*-glycosylation sites not encoded in the germline. This is an indication that they have been acquired by somatic hypermutation. The new *N*-glycosylation sites were found mainly in the framework region 3 (FR3) encoding structural integrity of immunoglobulins. A smaller fraction of the NXT/S motif was present in the complementary determining regions. Fab-hyperglycosylation has been described in other types of autoimmune diseases [72, 73]. Two recent studies [74, 75] found that anti-citrullinated protein antibodies (ACPA) in rheumatoid arthritis have a high frequency of *N*-glycans in the hypervariable domains. Similar to what was observed in MPO-ANCA patients, the vast majority of *N*-glycosylation sites in the Fab domain of ACPA were acquired by somatic hypermutations within FR3. In Sjögren’s syndrome [73], a higher prevalence of IgG sequences with *N*-linked glycosylation sites is seen when compared with healthy controls. It was suggested that B cell hyperproliferation and selection within the parotid gland may result from interactions of lectins present in the microenvironment that could confer a selective advantage to autoantibody-producing cells independent of conventional Ag binding at the CDRs [73]. In malignancy, differences in Fab glycosylation have been observed [76–81] that may be due to interactions between lectins and *N*-linked Fab glycans within lymphoid follicles [82, 83]. Together, these findings provide clues to a potential role for *N*-linked Fab glycans in conferring a selective advantage to autoantibody-producing B cells. So far, however, the physiological significance of ANCA Fab-hyperglycosylation remains to be elucidated, and additional studies are needed to definitively establish whether *N*-linked Fab glycosylation sites offers a selective advantage to ANCA-producing B cells.

A major limitation of this study is that it does not include data concerning Fab fragments glycosylation of anti-PR3 specific autoantibodies. The main reason is the prohibitive cost associated with the purification of anti-PR3 specific autoantibodies as commercially available purified PR3 is significantly more expensive than MPO. Results of this work suggest a clear need to develop an alternative protocol for ANCA purification and/or utilization of more sensitive mass spectrometers that would minimize antigen consumption.

In summary, this is the first study to demonstrate that significant differences exist between MPO-ANCA and PR3-ANCA diseases regarding the changes in amounts and types of glycans on the Fc portion of total IgGs with disease activity. Our results indicate that the prevalence of Fab glycosylation on anti-MPO IgGs is significantly increased when compared to anti-MPO-depleted IgGs. Given that aberrantly glycosylated IgG have different Fc-receptor and complement binding affinities [84, 85] and considering the impact of Fab glycosylation on stability, half-life and binding characteristics of antibodies and B cell receptors [34], differences in IgG Fc and/or Fab glycosylation may significantly contribute to differences in disease course or relapse rate observed between the two diseases. The identification of glycan traits that separate PR3-ANCA patients in active disease and remission suggests that these traits could be useful markers of disease activity and early relapse detection. Validation studies using a much larger cohort of patients would need to be carried out in a prospective fashion to support these findings.

## Supporting information

S1 Text Supplemental materials and methods. (DOCX)

S1 Table Compositions and calculated m/z values of the 14 most abundant tryptic glycopeptides of human IgG detected in the present study. (DOCX)

S2 Table Characteristics of patient groups and healthy controls for which the serum IgG Fc subclass specific glycosylation was determined. (DOCX)

S3 Table Pairwise comparison of IgG glycosylation between ANCA patients and controls. (DOCX)

S4 Table Pairwise comparison between IgG_1_ Fc glycosylation traits of ANCA patients and controls, stratified by disease. (DOCX)

S5 Table Correlation analysis between IgG_1_ and IgG_2/3_ Fc galactosylation. (DOCX)

S6 Table Correlation analysis between IgG Fc galactosylation and sialylation. (DOCX)

S7 Table Ability of galactose-derived glycan trait to distinguish active ANCA-associated vasculitis (ANCA) from remission or from healthy controls: receiver operating characteristic (ROC) analyses and likelihood ratios. (DOCX)

S8 Table Characteristics of MPO-ANCA positive patient group undergoing plasmapheresis included in this study. (DOCX)

S9 Table Correlation analysis between sialic content of affinity purified fractions and BVAS. (DOCX)

S10 Table Peptides containing *N*-glycosylation sites that were identified in the present work and comparison to most homologous germline-encoded sequences. (DOCX)

S1 Fig Correlation between IgG Fc glycan traits. A: correlation of the galactosylation of IgG_1_ and IgG_2/3_. B: correlation between IgG_1_ galactosylation and sialylation. C: correlation between IgG_2/3_ galactosylation and sialylation. Each glycosylation feature is expressed as a percentage of the total ion abundance of the 14 glycoforms analyzed in this study.
(DOCX)
