# Supplementary material for: Immunoglobulins G from patients with ANCA-associated vasculitis are atypically glycosylated in both the Fc and Fab regions and the relation to disease activity
Source: PLoS One. 2019 Feb 28;14(2):e0213215. doi: 10.1371/journal.pone.0213215 (PMC6395067; doi:10.1371/journal.pone.0213215)
Supplement: S1 Text — (DOCX) [file pone.0213215.s001.docx]

## S1 Text. Supplemental material and methods

### Polyclonal IgG purification

IgG was isolated from serum or plasma samples using protein AG agarose (**Santa Cruz Biotechnology,** Dallas, TX). Thawed plasma diluted 1:1 with 0.1 M sodium bicarbonate buffer, pH 8.3, was incubated with protein AG agarose beads for 3 hours at room temperature, in rotation. Beads were then washed with 10 column volumes of phosphate-buffered saline (PBS), pH 7.4, bound IgG was eluted with 0.1 M citric acid, pH 3.0, and directly neutralized with 0.1 M sodium bicarbonate buffer, pH 8.3. The resulting IgG solution was buffer-exchanged with PBS buffer using PD-10 gel filtration column (GE Healthcare, Pittsburgh, PA), concentrated using 30 KDa molecular weight cut off concentrator (Amicon, EMD Millipore, MA), and stored at 4°C. The purity of the resulting IgG preparations was assessed by sodium dodecyl sulfate-polyacrylamide gel electrophoresis (SDS-PAGE). Sample concentrations were measured by nanodrop (Thermo Scientific, Wilmington, DE), assuming a molar extinction coefficient of 198,560 M^-1^cm^-1^ at 280 nm [1].

### Generation of IgG-F(ab’)2 and IgG-Fc fragments

F(ab’)_2_ and Fc fragments were prepared by enzymatic digestion of purified IgG with recombinant *Streptococcus pyogenes* IdeS (Genovis, Cambridge, MA), a protease that cleaves all the human IgG subclasses within the lower hinge region [2]. Digestions were carried out with 20 U of enzyme per 80 µg IgG in phosphate-buffered saline, pH 7.4 for 2 hours at 37°C, and terminated by addition of the SDS-PAGE sample buffer. Fragment purity was checked SDS-PAGE followed by Coomassie brilliant blue staining.

### Pull-down experiments using SNA-coated agarose beads

Lectin affinity purifications were performed using *Sambucus nigra* agglutinin (SNA; Vector Laboratories, Burlingame, CA), which is specific for terminal sialic acid. The lectin affinity resin was equilibrated prior to use with 10 mM HEPES buffer, pH 7.5, containing 150 mM NaCl and 0.1 mM CaCl_2_ (HBS). All polyclonal IgG solutions used were buffer-exchanged into HBS and concentrated to a final concentration 0.1-0.3 mg/ml prior to lectin affinity purification. Purifications were performed in the form of pull-down assays whereby equal volumes of equilibrated SNA agarose resin and buffer-exchanged polyclonal IgG solutions were mixed together and incubated overnight at 4°C, in rotation. The beads were then pulled down by centrifugation (1000 rpm, 2 minutes), the supernatant was collected, and stored at 4°C. Prior to the elution of bound IgGs, the beads were washed 3 times with HBS. Bound IgGs were then eluted from the beads by incubating them with an equal volume of 0.5 M Lactose in HBS for 15 min at room temperature, in rotation. The beads were then pulled down again (1000 rpm, 2 minutes) and the supernatant that contains the eluted IgG was collected. Both the eluted material (Sialic-enrich IgG) and the supernatant (Sialic-depleted IgG) were buffer-exchanged into HBS, concentrated by ultrafiltration to 0.1-0.3 mg/ml, and stored at 4°C for subsequent analysis.

### Pull-down experiments using MPO-coated sepharose beads

Affinity isolation of anti-MPO autoantibodies was performed using NHS-activated sepharose beads coated with MPO. Briefly, 750 µg of MPO from human neutrophil (Lee Biosolutions, Maryland Heights, MO) was coupled to 500 µl of NHS-activated sepharose beads, according to manufacturer instruction (GE Healthcare). The remaining active groups on the beads were blocked with 1 M Tris buffer, pH 8.0, the coated beads were washed three times with 10 ml phosphate-buffered saline, pH 7.4, and incubated overnight at 4°C with 10 ml of purified IgG isolated from plasma sample (0.5 to 2 mg/ml equilibrated in 0.1 M sodium bicarbonate buffer, pH 8.3). The beads were then pulled down by centrifugation (1000 rpm, 2 minutes), and the supernatant was collected. Following the washing steps with 3 × 10 ml PBS, the bound IgGs were eluted from the beads with 0.1 M citric acid, pH 3.0, and directly neutralized with 0.1 M sodium bicarbonate buffer, pH 8.3. Both the eluted material (MPO-ANCA enriched IgG) and the supernatant (MPO-ANCA depleted IgG) were buffer-exchanged with PBS buffer, concentrated by ultrafiltration to 0.1-0.3 mg/ml, and stored at 4°C for subsequent analysis.

### Enzyme-linked lectin assay (ELLA)

Enzyme-linked lectin assays were performed using biotinylated SNA in a 96-well plate assay format (Greiner Bio-One, Monroe, NC) as previously described [3] with some modifications. Unfractionated polyclonal IgG or affinity-purified IgG glycovariants were first diluted with coating buffer [100 mM sodium carbonate buffer, pH 9.4] to a concentration of 1 µg/ml, and 200 µl of this dilution was applied to each well (0.2 µg of protein per well). Coated plates were blocked overnight with phosphate-buffered saline, pH 7.4, containing 0.5% polyvinyl alcohol, as suggested by Thompson *et al* [3]. The wells were washed three times with 50 mM Tris (pH 7.4), 150 mM NaCl, and 0.1% Tween 20 (TBS-Tween). Thereafter, 200 µl of 1 µg/ml solution of biotinylated *Sambucus nigra* lectin prepared in TBS-Tween was added to each well, and the plates were incubated for 1 h at 37°C. After three washes with TBS-Tween to remove unbound lectin, 200 µl of 1 µg/ml solution of streptavidin Alkaline phosphatase conjugate (Thermo Scientific), diluted in TBS-Tween, was added to each well, and the plates were incubated for 1 h at 37°C. After three additional washes, plates were developed by adding 200 µl of CDP-star chemiluminescence substrate (Roche, Indianapolis, IN) diluted 1:1000 in 50 mM Tris buffer, pH 9.6, to each well and the chemiluminescence signal was detected using a Tecan Infinite M200 microplate reader (Tecan US, Research Triangle Park, NC) and processed using the Xfluor™ software (Tecan US).

### Lectin blot analysis

Sialylation of unfractionated IgG isolated from plasma sample or of affinity-purified IgG glycovariants was assessed by SDS-PAGE followed by lectin blotting with biotinylated SNA as described by Käsermann *et al* [4] with modifications. Briefly, the indicated amounts of unfractionated IgG isolated from plasma sample or of affinity-purified IgG glycovariants were resolved by SDS-PAGE by using Novex 4-12% Bis-Tris polyacrylamide gel (Life Technologies, Grand Island, NY). Proteins were electroblotted onto nitrocellulose membranes using iBlot gel transfer device (Life Technologies). Membranes were blocked with 3% BSA in 100 mM sodium acetate buffer, pH 5.0 for 2 hours at room temperature, washed three times with TBS-Tween buffer, probed with biotinylated SNA lectin (1 µg/ml; Vector Laboratories, Burlingame, CA) for 30 minutes at RT, followed by three washes with TBS-Tween buffer and by incubation with a streptavidin alkaline phosphatase conjugate (1 µg/ml; Thermo Scientific) for 30 minutes at RT. After three additional washes with TBS-Tween buffer, the blots were developed by means of the Western blue stabilized substrate for alkaline phosphatase (Promega, Madison, WI). The relative intensity of bands was quantified by using the NIH ImageJ software.

### Anti-MPO ELISA

Detection of MPO-ANCA by a direct noncompetitive ELISA using purified human myeloperoxidase was performed as previously described [5] with modifications. High-binding 96-well ELISA microplates (Greiner Bio-One, Monroe, NC) were coated for 1 h at 37°C with 200 µl of 2.5 µg/ml purified human myeloperoxidase (Lee Biosolutions, Marylands Heights, MO) in 0.2 M sodium carbonate buffer, pH 9.4. Coated plates were blocked overnight with 0.2 M sodium carbonate buffer, pH 9.4 containing 4% fish gelatin and 0.05% Tween 20. The plates were washed three times with 0.05 M Tris buffer, pH 7.4, containing 0.2% fish gelatin, 0.05% Tween 20 (TB-Tween). Thereafter, 200 µl of 1 µg/ml solution of affinity-purified IgG samples diluted in TB-Tween was added and incubated for 1 h at 37°C. After three washes with TB-Tween, the secondary antibody, goat anti-human IgG-alkaline phosphatase (EMD Millipore, Darmstadt, Germany) diluted 1:10,000 in TB-Tween, was added and incubated for 1 h at 37°C. After three additional washes, the antigen-antibody complexes were detected using a chemiluminescence system and Xfluor Software (Tecan US), as described above.

### References

1. Howard, G.C. and M.R. Kaser, *MAKING AND USING ANTIBODIES A Practical Handbook Preface.* Making and Using Antibodies: A Practical Handbook, 2nd Edition, 2014: p. Vii-Vii.

2. von Pawel-Rammingen, U., B.P. Johansson, and L. Bjorck, *IdeS, a novel streptococcal cysteine proteinase with unique specificity for immunoglobulin G.* EMBO J, 2002. **21**(7): p. 1607-15.

3. Thompson, R., et al., *Optimization of the enzyme-linked lectin assay for enhanced glycoprotein and glycoconjugate analysis.* Anal Biochem, 2011. **413**(2): p. 114-22.

4. Kasermann, F., et al., *Analysis and functional consequences of increased Fab-sialylation of intravenous immunoglobulin (IVIG) after lectin fractionation.* PLoS One, 2012. **7**(6): p. e37243.

5. Franssen, C.F., et al., *Determinants of renal outcome in anti-myeloperoxidase-associated necrotizing crescentic glomerulonephritis.* J Am Soc Nephrol, 1998. **9**(10): p. 1915-23.
