# Supplementary material for: Immunoglobulins G from patients with ANCA-associated vasculitis are atypically glycosylated in both the Fc and Fab regions and the relation to disease activity
Source: PLoS One. 2019 Feb 28;14(2):e0213215. doi: 10.1371/journal.pone.0213215 (PMC6395067; doi:10.1371/journal.pone.0213215)
Supplement: S1 Table — (DOCX) [file pone.0213215.s002.docx]

### S1 Table. Compositions and calculated m/z values of the 14 most abundant tryptic glycopeptides of human IgG detected in the present study.

| Glycan  Species nb^a^ | Short Name | Glycoform Composition^b^ | IgG_1_  [M+2H]^2+^ | IgG_2/3_^c^  [M+2H]^2+^ |
| --- | --- | --- | --- | --- |
| 1 | G_0_ | H3N4 | 1244.498 | 1228.503 |
| 2 | G_0_F | H3N4F1 | 1317.527 | 1301.532 |
| 3 | G_1_ | H4N4 | 1325.524 | 1309.529 |
| 4 | G_0_N | H3N5 | 1346.037 | 1330.042 |
| 5 | G_1_F | H4N4F1 | 1398.553 | 1382.558 |
| 6 | G_2_ | H5N4 | 1406.550 | 1390.555 |
| 7 | G_0_FN | H3N5F1 | 1419.066 | 1403.071 |
| 8 | G_1_N | H4N5 | 1427.064 | 1411.069 |
| 9 | G_2_F | H5N4F1 | 1479.579 | 1463.584 |
| 10 | G_1_FN | H4N5F1 | 1500.093 | 1484.098 |
| 11 | G_2_N | H5N5 | 1508.090 | 1492.095 |
| 12 | G_1_FS | H4N4F1S1 | 1544.101 | 1528.106 |
| 13 | G_2_FN | H5N5F1 | 1581.119 | 1565.124 |
| 14 | G_2_FS | H5N4F1S1 | 1625.127 | 1609.132 |

^a^ N-glycan symbolic representation and numbering as in Fig 2.

^b^ H, hexose; N, HexNAc; F, fucose; S, *N*-acetyl neuraminic acid (sialic acid).

**^c^** IgG_2_ and IgG_3_ are not distinguished by the profiling method.
