# Supplementary material for: Immunoglobulins G from patients with ANCA-associated vasculitis are atypically glycosylated in both the Fc and Fab regions and the relation to disease activity
Source: PLoS One. 2019 Feb 28;14(2):e0213215. doi: 10.1371/journal.pone.0213215 (PMC6395067; doi:10.1371/journal.pone.0213215)
Supplement: S2 Table — (DOCX) [file pone.0213215.s003.docx]

### S2 Table. Characteristics of patient groups and healthy controls for which the serum IgG Fc subclass specific glycosylation was determined.

|  | PR3-ANCA |  | MPO-ANCA |  | Control |
| --- | --- | --- | --- | --- | --- |
| Age at disease onset (year) |  |  |  |  |  |
| Median | 44.1 |  | 52.4 |  | 47.8 |
| Range | 14.2-77.3 |  | 10.3-78.0 |  | 13.8-85.2 |
| Gender |  |  |  |  |  |
| Male:female ratio | 9 / 7 |  | 8 / 6 |  | 6 / 13 |
| Race or ethnic group |  |  |  |  |  |
| White | 14 |  | 9 |  | 16 |
| Black | 2 |  | 3 |  | 0 |
| Other | 0 |  | 2 |  | 3 |
| Disease |  |  |  |  |  |
| MPA | 8 |  | 7 |  |  |
| GPA | 7 |  | 2 |  |  |
| Renal-limited | 1 |  | 5 |  |  |
| Average peak serum creatinine |  |  |  |  |  |
| (mg/dl) | 3.19 |  | 2.74 |  |  |
| MPO-ANCA peak titer |  |  |  |  |  |
| (U/ml) | 4.6 |  | 89.2 |  |  |
| PR3-ANCA peak titer |  |  |  |  |  |
| (U/ml) | 123.6 |  | 10.1 |  |  |
|  |  |  |  |  |  |
