# Supplementary material for: Immunoglobulins G from patients with ANCA-associated vasculitis are atypically glycosylated in both the Fc and Fab regions and the relation to disease activity
Source: PLoS One. 2019 Feb 28;14(2):e0213215. doi: 10.1371/journal.pone.0213215 (PMC6395067; doi:10.1371/journal.pone.0213215)
Supplement: S3 Table — (DOCX) [file pone.0213215.s004.docx]

### S3 Table. Pairwise comparison of IgG glycosylation between AAV patients and controls

|  | *p* value ^a^ | | | | | | |
| --- | --- | --- | --- | --- | --- | --- | --- |
|  | PR3-ANCA | | |  | MPO-ANCA | | |
| Glycoforms | Active *vs* Remission | Active *vs* Control | Remission *vs* Control |  | Active vs Remission | Active vs Control | Remission vs Control |
| G_0_F | **0.0011** | **0.0001** | 0.2421 |  | 0.8824 | **0.0051** | **0.0048** |
| G_1_F | 0.0510 | **0.0031** | 0.2937 |  | 0.1145 | 0.2325 | **<0.0001** |
| G_2_F | **0.0107** | **0.0006** | 0.1567 |  | 0.8971 | **0.0018** | **0.0010** |
| G_0_ | 0.2901 | 0.2494 | 0.6477 |  | 0.4193 | 0.9927 | 0.6001 |
| G_1_ | **0.0404** | 0.293 | 0.8747 |  | 0.0956 | 0.3345 | 0.6644 |
| G_2_ | **0.0046** | 0.2627 | 0.7246 |  | 0.1473 | 0.1606 | 0.8151 |
| G_0_FN | 0.9239 | **0.0045** | **0.0036** |  | 0.2065 | **0.0019** | **0.0007** |
| G_1_FN | 0.0671 | 0.3111 | 0.8679 |  | 0.3094 | 0.8318 | 0.5221 |
| G_2_FN | 0.0506 | 0.3005 | 0.9338 |  | 0.4122 | 0.7824 | 0.8667 |
| G_0_N | 0.1726 | 0.2036 | 0.5936 |  | 0.9310 | 0.3225 | 0.1838 |
| G_1_N | 0.5286 | 0.6608 | 0.8101 |  | 0.5286 | 0.2496 | 0.4891 |
| G_2_N | **0.0341** | 0.1917 | 0.6408 |  | 0.2273 | 0.0856 | 0.2392 |
| G_1_FSA | 0.9621 | 0.5095 | 0.4446 |  | 0.1710 | 0.4565 | 0.7943 |
| G_2_FSA | 0.1466 | 0.0901 | 0.6999 |  | 0.7769 | 0.1028 | **0.0479** |
|  |  |  |  |  |  |  |  |
|  |  |  |  |  |  |  |  |

^a^ *p* values < 0.05 are highlighted in bold and considered significant.
