# Supplementary material for: Immunoglobulins G from patients with ANCA-associated vasculitis are atypically glycosylated in both the Fc and Fab regions and the relation to disease activity
Source: PLoS One. 2019 Feb 28;14(2):e0213215. doi: 10.1371/journal.pone.0213215 (PMC6395067; doi:10.1371/journal.pone.0213215)
Supplement: S4 Table — (DOCX) [file pone.0213215.s005.docx]

### S4 Table. Pairwise comparison between IgG_1_ Fc glycosylation traits of AAV patients and controls, stratified by disease

|  | *p* value | | | | | | | |
| --- | --- | --- | --- | --- | --- | --- | --- | --- |
|  | PR3-ANCA | | | |  | MPO-ANCA | | |
| Glycosylation traits | Active *vs* Remission ^a^ | Active *vs* Control ^b^ | Remission *vs* Control ^b^ | |  | Active vs Remission ^a^ | Active vs Control ^b^ | Remission vs Control ^b^ |
| Galactosylation | **0.0027** | **0.0001** | | 0.1604 |  | 0.9800 | **0.0019** | **0.0013** |
| Sialylation | 0.1749 | 0.1823 | | 0.8350 |  | 0.6234 | 0.1938 | 0.0696 |
| Fucosylation | 0.1019 | 0.4413 | | 0.9089 |  | 0.1705 | 0.4618 | 0.6941 |
| Bisection | 0.6245 | 0.4726 | | 0.3222 |  | 0.1492 | 0.4825 | 0.1232 |
|  |  |  | |  |  |  |  |  |
|  |  |  | |  |  |  |  |  |

^a^ *p* values calculated using Wilcoxon matched-pairs signed rank test. *p* values < 0.0062 (Bonferroni corrected for 8 tests) are highlighted in bold and considered significant.

^b^ *p* values calculated using two-sided Mann-Whitney test. *p* values < 0.0031 (Bonferroni corrected for 16 tests) are highlighted in bold and considered significant.
