# Supplementary material for: Immunoglobulins G from patients with ANCA-associated vasculitis are atypically glycosylated in both the Fc and Fab regions and the relation to disease activity
Source: PLoS One. 2019 Feb 28;14(2):e0213215. doi: 10.1371/journal.pone.0213215 (PMC6395067; doi:10.1371/journal.pone.0213215)
Supplement: S5 Table — (DOCX) [file pone.0213215.s006.docx]

### S5 Table. Correlation analysis between IgG_1_ and IgG_2/3_ Fc galactosylation.

|  | Pearson r ^a^ | *p* value ^b^ |
| --- | --- | --- |
| Healthy Control | 0.8916 | **< 0.0001** |
| PR3-ANCA | 0.5702 | **0.0010** |
| MPO-ANCA | 0.5682 | **0.0011** |
| All samples | 0.6849 | **< 0.0001** |

**^a^** Two-tailed Pearson correlation analysis.

**^b^** *p* values < 0.05 are highlighted in bold and considered significant.
