# Supplementary material for: Immunoglobulins G from patients with ANCA-associated vasculitis are atypically glycosylated in both the Fc and Fab regions and the relation to disease activity
Source: PLoS One. 2019 Feb 28;14(2):e0213215. doi: 10.1371/journal.pone.0213215 (PMC6395067; doi:10.1371/journal.pone.0213215)
Supplement: S6 Table — (DOCX) [file pone.0213215.s007.docx]

### S6 Table. Correlation analysis between IgG Fc galactosylation and sialylation.

| Subclass^a^ | Sample | Pearson r ^b^ | *p* value ^c^ |
| --- | --- | --- | --- |
| IgG_1_ | Healthy Control | 0.6815 | **0.0013** |
|  | PR3-ANCA | 0.7875 | **< 0.0001** |
|  | MPO-ANCA | 0.5848 | **0.0007** |
|  | All samples | 0.6832 | **< 0.0001** |
| IgG_2/3_^d^ | Healthy Control | 0.7306 | **0.0004** |
|  | PR3-ANCA | 0.8032 | **<0.0001** |
|  | MPO-ANCA | 0.8616 | **<0.0001** |
|  | All samples | 0.8142 | **<0.0001** |

**^a^** The IgG_4_ glycopeptides were not analyzed due their low abundance.

**^b^** Two-tailed Pearson correlation analysis.

**^c^** *p* values < 0.05 are highlighted in bold and considered significant.

**^d^** IgG_2_ and IgG_3_ are not distinguished by the profiling method.
