# Supplementary material for: Immunoglobulins G from patients with ANCA-associated vasculitis are atypically glycosylated in both the Fc and Fab regions and the relation to disease activity
Source: PLoS One. 2019 Feb 28;14(2):e0213215. doi: 10.1371/journal.pone.0213215 (PMC6395067; doi:10.1371/journal.pone.0213215)
Supplement: S7 Table — (DOCX) [file pone.0213215.s008.docx]

### S7 Table. Ability of galactose-derived glycan trait to distinguish active ANCA-associated vasculitis (ANCA) from remission or from healthy controls: receiver operating characteristic (ROC) analyses and likelihood ratios.

|  | Active AAV versus remission | | | | |  | Active AAV versus controls | | | | |
| --- | --- | --- | --- | --- | --- | --- | --- | --- | --- | --- | --- |
| Disease | AUC^a^ | OCP^b^ | Sensitivity^c^ | Specificity^d^ | LR^e^ |  | AUC^a^ | OCP^b^ | Sensitivity^c^ | Specificity^d^ | LR^e^ |
| PR3-ANCA | 0.78 | 32 | 44 (20-70) | 87 (62-98) | 3.50 |  | 0.86 | 34 | 63 (35-85) | 95 (74-100) | 11.90 |
| MPO-ANCA | 0.51 | 23 | 14 (2 - 43) | 86 (57-98) | 1.00 |  | 0.80 | 33 | 57 (29-82) | 95 (74-100) | 10.86 |

^a^ AUC, area under the ROC curve. An AUC of 1 indicates perfect discrimination between groups; an AUC of 0.5 indicates no discrimination.

^b^ OCP, optimal cut-off point, which is the maximum sum of sensitivity and specificity.

^c^ Sensitivity at OCP. 95% confidence intervals are shown.

^d^ Specificity at OCP. 95% confidence intervals are shown.

^e^ LR, positive likelihood ratio at OCP.
