# Supplementary material for: Immunoglobulins G from patients with ANCA-associated vasculitis are atypically glycosylated in both the Fc and Fab regions and the relation to disease activity
Source: PLoS One. 2019 Feb 28;14(2):e0213215. doi: 10.1371/journal.pone.0213215 (PMC6395067; doi:10.1371/journal.pone.0213215)
Supplement: S8 Table — (DOCX) [file pone.0213215.s009.docx]

### S8 Table. Characteristics of MPO-ANCA positive patient group undergoing plasmapheresis included in this study.

| Age at disease onset (year) |  |
| --- | --- |
| Median | 56.2 |
| Range | 18.0-82.0 |
| Gender |  |
| Male:female ratio | 8 / 10 |
| Male (%) | 44.4 |
| Race or ethnic group |  |
| White | 9 |
| Black | 7 |
| Other | 1 |
| Disease |  |
| MPA | 5 |
| GPA | 5 |
| EGPA | 1 |
| Renal-limited | 4 |
