# Supplementary material for: Immunoglobulins G from patients with ANCA-associated vasculitis are atypically glycosylated in both the Fc and Fab regions and the relation to disease activity
Source: PLoS One. 2019 Feb 28;14(2):e0213215. doi: 10.1371/journal.pone.0213215 (PMC6395067; doi:10.1371/journal.pone.0213215)
Supplement: S9 Table — (DOCX) [file pone.0213215.s010.docx]

### S9 Table. Correlation analysis between sialic content of affinity purified fractions and BVAS ^a^.

| Fraction | Pearson r ^b^ | | *p* value ^c^ | |
| --- | --- | --- | --- | --- |
| Sample before fractionation (IN) | | 0.35 | | 0.22 |
| Anti-MPO enriched (BD) | | 0.39 | | 0.17 |
| Anti-MPO depleted (UB) | | 0.33 | | 0.24 |

**^a^** The affinity purified fractions were separated using antigen-coated beads. Sialic acid content was evaluated by enzyme linked lectin assay using SNA lectin. BVAS = Birmingham Vasculitis Activity Score.

**^b^** Two-tailed Pearson correlation analysis.

**^c^** *p* values < 0.05 are considered significant.
