# Supplementary material for: Immunoglobulins G from patients with ANCA-associated vasculitis are atypically glycosylated in both the Fc and Fab regions and the relation to disease activity
Source: PLoS One. 2019 Feb 28;14(2):e0213215. doi: 10.1371/journal.pone.0213215 (PMC6395067; doi:10.1371/journal.pone.0213215)
Supplement: S10 Table — (DOCX) [file pone.0213215.s011.docx]

S10 Table. Peptides containing *N*-glycosylation sites that were identified in the present work and comparison to most homologous germline-encoded sequences. The table lists all the *N*-glycosylation sites observed in the heavy and light chain variable regions. Comparison between *de novo* and germline sequences shows that the glycosylation sites detected by *de novo* sequencing are generally absent in the germline sequences.  New glycosylation sites were identified in the Framework 1 and 3 and CDR 1 and 2 regions of the Heavy chain variable region, and in the Framework 1 and 3 and CDR 1 regions of the light chain variable region.

### S10 Table (cont.).

### S10 Table (cont.).

### S10 Table (cont.).

### S10 Table (cont.).

^a^Heavy-chain and light-chain V region sequences are aligned to their respective germline sequences using IMGT/DomainGapAlign Tool [1].

^b^The consensus motif for *N*-linked glycosylation (NXS/T sequon) is boxed and the asparagine residues to which carbohydrate is linked are shaded in orange. Amino acids divergent from IMGT database top-matched germline sequence are shown in red. I to L mismatches are shaded in blue. The two residues cannot be directly distinguished in mass spectrometry since they have the same mass. PEAKS *de novo* sequencing software uses L to represent both I and L.

^c^Dots in germline sequences indicate sequence identity between the *de novo* query and the germline sequence whereas amino acids mismatches are indicated as text and shaded in grey.

^d^Top matched germline V genes and alleles using IgBLAST search algorithm developed at NCBI [2].

## References

1. Ehrenmann, F., et al., IMGT/3Dstructure-DB and IMGT/DomainGapAlign: a database and a tool for immunoglobulins or antibodies, T cell receptors, MHC, IgSF and MhcSF. Nucleic Acids Res., 2010. 38: D301–D307

2. Ye, J., et al., *IgBLAST: an immunoglobulin variable domain sequence analysis tool.* Nucleic Acids Res., 2013. 41: W34-W40
