# Supplementary figures and images for: Immunoglobulins G from patients with ANCA-associated vasculitis are atypically glycosylated in both the Fc and Fab regions and the relation to disease activity
Source: PLoS One. 2019 Feb 28;14(2):e0213215. doi: 10.1371/journal.pone.0213215 (PMC6395067; doi:10.1371/journal.pone.0213215)

### S1 Fig. Correlation between IgG Fc glycan traits.

**
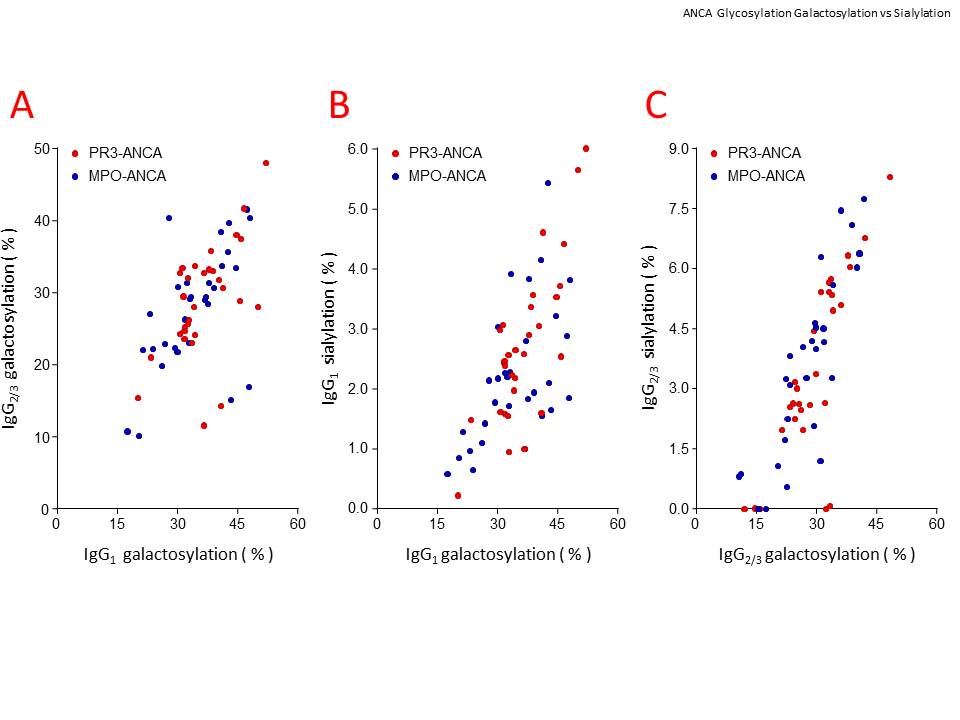
**

Supplement: S1 Fig — A: correlation of the galactosylation of IgG1 and IgG2/3. B: correlation between IgG1 galactosylation and sialylation. C: correlation between IgG2/3 galactosylation and sialylation. Each glycosylation feature is expressed as a percentage of the total ion abundance of the 14 glycoforms analyzed in this study. (DOCX) [file pone.0213215.s012.docx]
